# Influence of Baseline Kidney Function on Patient and Kidney Outcomes in Patients with COVID-19: A Multi-National Observational Study

**DOI:** 10.3390/jcm14041212

**Published:** 2025-02-12

**Authors:** Harin Rhee, Etienne Macedo, Gary Cutter, Eric Judd, Sreejith Parameswaran, Elizabeth Maccariello, Wen-Jiun Liu, Nicholas M. Selby, Josée Bouchard, Guillermo Garcia-Garcia, Javier A. Neyra, Yadla Manjusha, Josephine Abraham, Kent Doi, Guillermo Villamizar, Abdias Hurtado, Ravindra L. Mehta

**Affiliations:** 1 Department of Medicine, University of California San Diego, San Diego, CA 92093, USA; rheeharin@pusan.ac.kr (H.R.);; 2 Department of Medicine, Pusan National University School of Medicine, Busan 49241, Republic of Korea; 3 Department of Biostatistics, University of Alabama, Birmingham, AL 35294, USA; 4 Department of Medicine, Division of Nephrology, University of Alabama, Birmingham, AL 35294, USA; 5 Department of Medicine, Jawaharlal Institute of Postgraduate Medical Education & Research Pondicherry, Pondicherry 605006, India; 6 Department of Medicine, IDOR—D’Or Institute for Research and Education, Rede D’Or, Rio de Janeiro 2281-100, Brazil; 7 Department of Medicine, Hospital Sultanah Aminah, Johor Bahru 80100, Malaysia; plnjl@yahoo.com; 8 Centre for Kidney Research and Innovation, Academic Unit of Translational Medicinal Sciences, University of Nottingham, Nottingham NG7 2RD, UK; 9 Department of Medicine, University Hospitals of Derby and Burton NHS Foundation Trust, Derby DE22 3NE, UK; 10 Department of Medicine, Sacré-Coeur de Montreal Hospital, Montreal, QC H4J 1C5, Canada; 11 Centro Universitario de Ciencias de la Salud, Universidad de Guadalajara, Guadalajara 44340, Mexico; ggarcia1952@gmail.com; 12 Department of Medicine, Division of Nephrology, Bone and Mineral Metabolism, University of Kentucky, Lexington, KY 40508, USA; 13 Department of Medicine, Gandhi Medical College, Hyderabad 500003, India; 14 Department of Medicine, University of Utah Medical Center, Salt Lake City, UT 84112, USA; jo.abraham@hsc.utah.edu; 15 Department of Emergency and Critical Care Medicine, The University of Tokyo, Tokyo 113-8654, Japan; 16 Department of Medicine, Clinica Davila, Santiago 8431657, Chile; 17 Department of Medicine, Hospital Nacional Arzobispo Loayza, Lima 15082, Peru

**Keywords:** acute kidney injury, acute kidney disease, chronic kidney disease, no kidney disease, COVID-19

## Abstract

**Background/Objectives**: Acute kidney injury (AKI) is a common complication of coronavirus disease-19 (COVID-19), but the impact of baseline kidney function and care processes on outcomes is not well understood. We hypothesized that baseline kidney health status may influence courses and outcomes of AKI. **Methods**: This is a multinational, multicenter, retrospective cohort study. We included hospitalized adult COVID-19 patients with kidney disease (AKI, end-stage kidney disease (ESKD), chronic kidney disease (CKD), or kidney transplant (KT) recipients) from 1 January 2020 to 31 March 2022, across 52 centers in 23 countries. Patients with no prior kidney function information were classified as acute kidney disease (AKD) if estimated glomerular filtration rate (eGFR) at admission was <60 mL/min/1.73 m^2^ and as no known kidney disease (NKD) if eGFR was ≥60 mL/min/1.73 m^2^. We defined combined outcome as death or non-kidney recovery at hospital discharge. Multivariable binary regression models were applied. **Results**: Among 4158 patients, 882 had ESKD, and 3038 developed AKI. AKI patients were categorized as NKD (31.8%), AKD (38.6%), CKD (23.3%), and KT recipients (3.3%). NKD patients had higher AKI severity and more intensive care unit care needs. In the multivariable analyses, the risk of the combined outcome was higher in AKD (OR 1.459 [1.061, 2.005]) or CKD (OR 1.705 [1.206, 2.410]) patients, although the risk of in-hospital mortality was similar to NKD. Among the survivors at hospital discharge, the risk of partial or non-recovery was higher in CKD (OR 5.445 [3.864, 7.672]) or KT recipients (OR 4.208 [2.383, 7.429]) compared to NKD. These findings were consistent across income categories. **Conclusions**: Among AKI patients with COVID-19, nearly two-thirds had underlying kidney dysfunction, with 55% identified as having baseline AKD, which had higher risk of death or non-kidney recovery at discharge compared to NKD.

## 1. Introduction

The coronavirus disease-19 (COVID-19) pandemic, caused by the severe acute respiratory syndrome coronavirus 2 (SARS-CoV-2), has led to over 767 million infections and more than 6.9 million deaths globally [1]. Acute kidney injury (AKI) is a common complication in severe COVID-19 cases, occurring in 40–50% of patients and significantly increasing mortality rates [2,3]. The severity and need for dialysis in COVID-19-related AKI are higher compared to non-COVID-19 AKI [4]. Acute tubular necrosis is the most common pathologic finding in the kidneys of patients with COVID-19 and AKI, followed by collapsing glomerulopathy or thrombotic microangiopathy [5,6]. Systemic or local immune response to COVID-19 is thought to cause AKI [7]. Although several researchers have identified viral particles in the kidney tissue, whether direct viral invasion triggers AKI remains controversial [8,9].

Patients with chronic kidney disease (CKD) are especially vulnerable to developing AKI during COVID-19 and have a higher risk of non-recovery [10,11,12]. These patients also experience a faster decline in kidney function post hospitalization [13]. AKI mortality rates vary widely and could be influenced by a country’s income level and health expenditure [14]. Health disparities impact incident AKI and CKD development and progression, potentially affecting COVID-19-associated kidney disease outcomes, although this relationship is not well described [15].

This study evaluates the epidemiology of COVID-19 patients with acute or chronic kidney diseases across multiple centers worldwide. We hypothesized that baseline kidney function and care processes influence patient outcomes. We aimed to understand the relationship between kidney disease and COVID-19 to inform prevention, diagnosis, and treatment strategies.

## 2. Methods

### 2.1. Design and Setting

This global multicenter retrospective observational cohort study enrolled COVID-19-positive patients over 18 years old and with kidney impairment in 52 centers across 23 countries from January 2020 to March 2022 (ClinicalTrials.gov identifier NCT04491227). Physicians recruited through open invitations identified hospitalized patients with confirmed COVID-19 and acute or chronic kidney disease. The study was conducted according to the guidelines of the Declaration of Helsinki. The study protocol was approved by the institutional review board at University of California, San Diego, with a waiver of informed consent due to the observational nature of the study (45 CFR 46.104(d), category 4; Health Insurance Portability and Accountability Act (HIPAA) Privacy Rule, 45 CFR 164 Section 512(I), 13 April 2020). Ethical approval was obtained for each participating country and site according to local requirement. Participation was voluntary and without financial incentive. The patients included met criteria for AKI, had known CKD with a baseline estimated glomerular filtration rate (eGFR) < 60 mL/min/1.73 m^2^, were on chronic dialysis (ESKD), or were kidney transplant (KT) recipients.

### 2.2. Data Collection

Pre-existing patient data, standard-of-care labs, vital signs, and routine clinical measurements were recorded at hospital admission and weekly until discharge. Data were submitted using a customized CDC-EPI Info mobile application, which synchronized automatically to a central database at University of California, San Diego [16]. Collected data included demographics, comorbidities, longitudinal lab values, physiologic parameters, volume status, pre-hospitalization medications, drugs given during the hospitalizations, organ support (e.g., ventilator or dialysis), kidney recovery, and survival at discharge. A complete list of variables is provided in Table S2.

### 2.3. Definitions

Chronic dialysis-dependent patients were classified as having ESKD. Baseline kidney health was determined by reviewing available data and serum creatinine (SCr) values at hospital admission. Baseline SCr was defined as the latest measured SCr within 3–12 months prior to admission. If unavailable, the admission SCr was used. Baseline eGFR was calculated using the Chronic Kidney Disease Epidemiology Collaboration (CKD-EPI) equation [17]. Patients were classified as CKD if they had a known diagnosis, markers of kidney damage for more than 3 months, or eGFR < 60 mL/min/1.73 m^2^ for more than 3 months [18,19]. Patients with no prior kidney function information were classified as acute kidney disease (AKD) if eGFR at admission was <60 mL/min/1.73 m^2^ [19,20] and as no known kidney disease (NKD) if eGFR was ≥60 mL/min/1.73 m^2^.

AKI was confirmed for patients who met at least one of the modified KDIGO criteria [21]: (1) SCr change (increase or decrease) > 0.3 mg/dL within 48 h or >50% within 7 days; (2) urine output <400 mL/day. Reference SCr was considered the sCr level at admission. AKI severity was assessed at diagnosis and peak using KDIGO stages. AKI within 48 h of admission was classified as community-acquired (CA) AKI, while hospital-acquired (HA) AKI referred to AKI developing later.

Kidney recovery in AKI patients was assessed at hospital discharge as (1) complete recovery (SCr returned to baseline or lower), (2) partial recovery (SCr lower than peak but higher than baseline), and (3) non-recovery (dialysis dependence at discharge). CKD progression in CKD or KT patients without AKI was defined as a 50% or more increase in serum creatinine from baseline. Details of the definition are summarized in Table S3.

### 2.4. Country Income Groupings

Countries were classified into three income groups based on 2019 gross national income (GNI) per person using the World Bank Atlas method: high-income countries (HICs) (GNI > USD 12,535), upper-middle-income countries (UMICs) (USD 4046 < GNI ≤ USD 12,535), and lower-middle-income countries (LMICs) (USD 1036 < GNI ≤ USD 4046).

### 2.5. Outcomes

Outcomes included AKI patterns (community- or hospital-acquired), oliguria status, AKI severity and duration, acute illness course, processes of care, length of hospital stay (LOS), in-hospital death, kidney recovery, and combined outcome of death or non-recovery at discharge. Outcomes were compared across different kidney health-status groups and income groups.

### 2.6. Statistical Analysis

Continuous variables were presented as mean ± standard deviation (SD) or median with inter quartile range (IQR), based on data normality. The Kolmogorov–Smirnov test checked data normality. Differences in continuous variables were compared using ANOVA or Kruskal–Wallis tests. Categorical variables are presented as numbers and proportions and were compared using the chi-square test. Post hoc tests with Bonferroni adjustment were performed for both categorical and non-categorical variables.

Unadjusted and adjusted models (for age and sex) were used. Multivariable binary regression analyses assessed the impact of kidney health status on in-hospital mortality and kidney recovery. Variables with *p* < 0.20 in univariable analysis were included in multivariable regression using a backward stepwise approach to develop the final model (*p* < 0.10). Hazard ratios with 95% CIs were reported. All tests were two-sided, with *p* < 0.05 considered significant after the Bonferroni adjustment. Data were analyzed using IBM SPSS (version 26.0, SPSS Inc., Chicago, IL, USA).

## 3. Results

### 3.1. Baseline Characteristics

**Figure 1:**
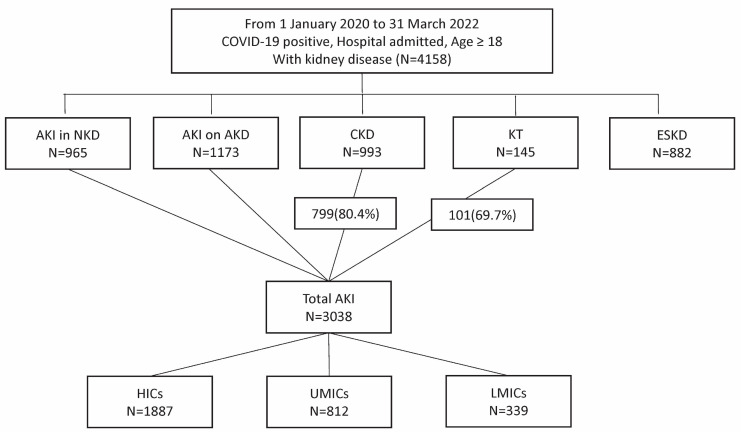
Flow diagram of the patients. Abbreviations: AKI, acute kidney injury; NKD, no kidney disease; AKD, acute kidney disease; CKD, chronic kidney disease; KT, kidney transplant recipients; ESKD, end-stage kidney disease; HICs, high-income countries; UMICs, upper-middle-income countries; LMICs, lower-middle-income countries.

**Figure 2:**
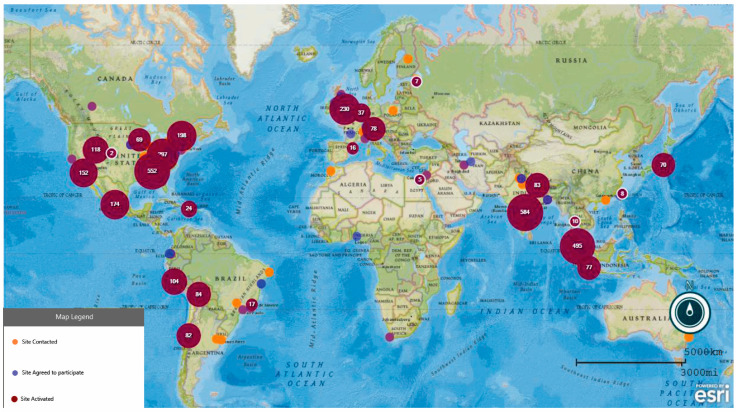
The distribution of the patients included. Note: This figure was plotted using a tool supported by CDC-EPI Info for Windows.

From 1 January 2020 to 31 March 2022, 4158 patients with kidney disease and COVID-19 were enrolled. This included 882 with ESKD, 993 with CKD, 145 KT recipients, 1173 with AKD, and 965 with NKD. AKI was identified in 3038 patients, including 799 with CKD and 101 KT recipients (Figure 1). Patients were from high-income countries (53.5%), upper-middle-income countries (28.7%), and lower-middle-income countries (17.8%) (Figure 2).

The median age was 64 years (IQR 52–74), with 63.5% male and 74.7% non-White (Table 1 and Table S4). Patients had a median of two comorbidities, with hypertension being the most frequent. AKI patients were more common in HICs (62.1%) and had higher rates of lung or heart disease and use of beta-blockers, diuretics, angiotensin-converting enzyme (ACE) inhibitors, angiotensin receptor blockers (ARBs), and anticoagulants. ESKD patients were more common in Asia (65.3%) and LMICs (38.8%), were younger, and had higher rates of diabetes and hypertension.

The main reasons for hospitalization were respiratory issues (61.1%), non-respiratory infection (39.8%), and worsening renal disease (23.5%). During hospitalization, 45.3% required intensive care unit (ICU) admission, 38.3% needed ventilation support, and 23.2% received both ventilation and dialysis care, with these needs most frequent among AKI patients.

### 3.2. Differences in Patient Characteristics, Course, and Care Processes by Baseline Kidney Health Status and Country Strata Among AKI Patients

In the AKI cohort, 965 (31.8%) had NKD, 1173 (38.6%) had AKD, 799 (26.3%) had CKD, and 101 (3.3%) were KT recipients. NKD patients were younger, had fewer comorbidities, and used fewer medications compared to AKD and CKD patients. Respiratory diagnoses and non-respiratory infections were more common in NKD patients, while worsening renal function and metabolic complications were more frequent in AKD and CKD patients. AKD patients had higher rates of diabetes, cardiovascular disease, and statin and beta-blocker use compared to NKD patients but lower than CKD patients.

Patients from HICs were older, had more chronic lung disease or heart failure, and used more beta blockers, statins, and diuretics compared to those from UMICs or LMICs. Severe hospital admission causes, like shock, sepsis, infection, respiratory conditions, and metabolic issues, were more common in UMICs and LMICs than in HICs.

AKI was diagnosed in 6.9% of cases based on urine output criteria and in 60.1% of cases based on an increase in serum creatinine, while 33.0% showed a decrease in serum creatinine. NKD patients were less likely to be oliguric at admission and were more frequently diagnosed with AKI by an increased serum creatinine compared to other groups (Table 2).

Among 2813 patients (92.6%) with available data, hospital-acquired AKI (HA-AKI) was most common in NKD patients (69.5%). At the same time, community-acquired AKI (CA-AKI), oliguria, and dehydration were more frequent in AKD or CKD patients (Table 2). At AKI diagnosis (in 2607 patients, 85.8%), most were stage 1 (71.1%). Kidney replacement therapy (KRT) was required at diagnosis in 341 patients (13.1%). At peak AKI severity, 51.4% were stage 2 or higher, with 32.1% requiring KRT. ICU care and ventilator support were needed for 1593 patients (52.4%) and 1379 patients (45.6%), respectively. NKD patients had higher AKI severity at diagnosis and peak, with greater ICU care, ventilator support, and trial participation compared to AKD or CKD patients (Table 2). In HICs, continuous kidney replacement therapy (CKRT) was the most common dialysis modality, while over 80% of patients in LMICs received intermittent hemodialysis. Investigational trial enrollment was 15.9% in HICs, 1.0% in UMICs, and 0.4% in LMICs. Differences in AKI characteristics or care processes by baseline kidney function status were consistent regardless of national income status (Table S5).

### 3.3. Patient Outcomes by Kidney Health Status

The overall median LOS in hospital was 12 days (range: 7 to 21 days), with AKI patients having a longer LOS of 13 days (range: 7 to 23 days). Among AKI patients, those with NKD had the longest LOS at 17 days (range: 9 to 30 days) (Table 2).

The in-hospital mortality rate was 38.4%, which was higher in AKI patients at 42.5% (Table 1). NKD (47.1%) and AKD (42.4%) patients had the highest mortality rates, while the combined outcome of death or non-recovery was similar to that in CKD or KT recipients (Table 2).

Of the 1688 AKI survivors, 655 (38.8%) experienced partial or non-recovery of kidney function, which was more common in CKD (61.1%) and KT recipients (56.6%) compared to NKD (26.7%) and AKD patients (29.9%).

### 3.4. Prediction of Hospital Mortality, Combined Outcome, and Non-Recovery

Higher in-hospital mortality was associated with older age, congestive heart failure (CHF), liver disease, shock at admission, ICU admission, and ventilator support with or without KRT. Protective factors included being of Black race, statin use, participation in investigational trials, and absence of AKI. The process of care influenced mortality differently by national income status, with higher odds of death in UMICs and LMICs compared to HICs (Table S6). The risk of death in AKI patients was similar to that in patients with ESKD, whereas CKD or KT recipients without AKI had a significantly lower risk of death compared to those with ESKD (Table 3).

Higher AKI stage was a specific risk factor for in-hospital mortality in AKI patients (Table S7). The crude and age–sex-matched risk of death was highest in NKD patients, but after adjusting for various factors, there was no significant difference in mortality between AKD, CKD, KT, and NKD patients (Table 3). Risk factors for the combined outcome of death or non-recovery included older age, CHF, higher AKI stage, ventilator or KRT use, and baseline kidney dysfunction (AKD, CKD, and KT). Statin use was associated with better outcomes (Table S7). The odds of the combined outcome were higher in AKD and CKD patients compared to NKD patients (Table 3).

National income status alone was not directly associated with outcomes. Adjusted models showed no significant difference in prognosis between income groups after accounting for multiple factors (Table S8). Among AKI survivors, non-recovery risk factors included living in LMICs, HA-AKI, higher AKI stage, CKD, and being a KT recipient (Table 3 and Table S9).

## 4. Discussion

This multinational, multicenter study provides new insights into the influence of baseline kidney health status and care processes on COVID-19 outcomes in patients with kidney disease. Unlike previous research focused on AKI or CKD based on KDIGO criteria, this study includes patients with AKD, a condition often unrecognized but linked to higher risks of CKD progression, ESKD, and death [2,4,20,22,23]. Previous studies have also shown that patients with baseline CKD or AKD have higher incidences of AKI and mortality in non-COVID contexts [24,25]. We compared COVID-19 patients based on their kidney health at hospital admission. Notably, nearly two-thirds of non-ESKD or KT patients had underlying kidney dysfunction, with 55% identified as having baseline AKD. The study found that kidney health status influenced AKI development timing, AKI stage, kidney recovery, and KRT use.

In contrast to prior studies, we included oliguria and decline in serum creatinine in addition to increase in serum creatinine as markers of AKI. Oliguria was observed in 6.9% of patients at AKI diagnosis, particularly in those with decreased kidney reserve and better access to care, such as AKD or CKD patients from HICs. Including decreasing serum creatinine criteria in AKI diagnosis identified 33.0% more patients, aligning with ISARIC findings [26]. These patients had similar clinical characteristics to traditional AKI patients and generally worse outcomes than non-AKI patients [26].

Non-CKD patients were categorized into AKD and NKD groups based on eGFR at admission. AKD patients had more frequent AKI stage 1 and required less ICU admission, ventilator support, or KRT. Despite these differences, in-hospital mortality odds were similar between AKD and NKD patients, though AKD patients had a 50% higher risk of combined in-hospital mortality or non-kidney recovery. This may be due to decreased kidney function reserve, comorbidities, and polypharmacy in AKD patients.

Prior studies have shown differences in outcomes between CA and HA AKI in COVID-19 but did not distinguish the influence of baseline kidney health. Among AKD, CKD, or KT patients, 80% of AKIs were community-acquired, while over 50% of AKIs in NKD patients were hospital-acquired. AKD patients had a higher risk of CA-AKI than NKD patients, highlighting the importance of distinguishing baseline AKD in the differentiation of AKI phenotypes.

The distinction between CA- and HA-AKI is important, as it influences patient outcomes. CA-AKI is more prevalent in patients with multiple comorbidities, including CKD, while HA-AKI is more common in younger patients with severe multi-organ disease during COVID-19 [27,28]. Bell et al. [29] found higher mortality in HA-AKI compared to CA-AKI, though CA-AKI still had elevated mortality compared to patients without AKI. Our study found non-recovery of kidney function more common in non-NKD patients. After adjusting for factors like AKI recovery and baseline kidney function, mortality risk between HA-AKI and CA-AKI became comparable, suggesting that baseline kidney health significantly affects outcomes.

Pre-existing ESKD has been a significant predictor of in-hospital mortality in COVID-19 patients. A New York study reported an odds ratio of 1.37 for in-hospital mortality in ESKD patients [30]. Data from the ERA-EDTA registry indicated a 20% COVID-19-attributable mortality rate in ESKD patients compared to a matched historical control [31]. Our study aligns with these findings, showing an increased risk of mortality in ESKD patients compared to CKD or KT recipients without AKI. Interestingly, ESKD patients were less likely to require ICU admission, and mortality rates were lower than those with AKI. The majority were managed with IHD, and most survivors were discharged to home. ESKD patient numbers were 3–5 times higher in UMICs and LMICs than in HICs, with mortality differing based on country income. Mortality in ESKD patients was twice as high in LMICs, possibly due to challenges in quarantine measures for dialysis patients and limited access to investigational drugs like remdesivir or tocilizumab [32,33,34].

Our findings on baseline kidney health status were consistent across national income levels, but significant differences were observed in clinical courses, treatment levels, and outcomes based on country income. HA-AKI or advanced-stage AKI at diagnosis was more common, ICU admissions and life-supporting device utilization were more frequent, and death or combined outcomes were significantly higher in UMICs than in HICs. The use of ventilators and KRT was linked to higher death odds, especially in lower-income countries, likely due to disparities in care access and fewer clinical trial opportunities. Clarifying partial or non-kidney recovery in patients with baseline kidney dysfunction versus new-onset CKD or CKD progression after AKI is crucial, typically requiring a three-month interval. The study found CKD progression in CKD or KT recipients without AKI as a 50% serum creatinine increase from baseline, occurring in about 3% of the population, suggesting COVID-19 hospital admissions may influence CKD progression.

The present study’s strengths include its multinational, multicenter design, providing a comprehensive view of COVID-19’s impact on kidney dysfunction across diverse healthcare systems. The CDC-EPI Info mobile app ensured efficient data collection with minimal missing data over two years. Multiple time points throughout the pandemic allowed for trend analysis in patient characteristics, outcomes, and management strategies.

However, the convenience cohort design may introduce selection bias and not fully represent the affected population. Changes in COVID-19 subtypes with different severity by time period, details of investigational trial drugs, and vaccination status were not reflected in this manuscript. Weekly data collection could miss daily fluctuations, and the lack of specific medication dosing information limits the assessment of their impact on outcomes. Effects of sodium-glucose cotransporter 2 inhibitors or glucagon-like peptide-1 receptor agonists on kidney outcomes of COVID-19 patients were not considered. It is unclear whether the AKD patients had unrecognized CKD or had developed AKI in the community prior to hospitalization.

## 5. Conclusions

This study highlights the importance of baseline kidney function in managing COVID-19 patients with kidney dysfunction, emphasizing the need for targeted interventions and addressing healthcare disparities to ensure equitable care. We especially focused on the significance of baseline AKD in the course and outcomes of AKI. Compared to the patients with NKD, AKD patients had more comorbidities and took more home medications. CA-AKI and dehydration were more common at presentation. AKI stage at diagnosis was relatively mild, and ICU admission, ventilator, or dialysis utilization was less frequent than in NKD. Nevertheless, the odds of in-hospital mortality or non-kidney recovery were 1.5-fold higher than in NKD. The characteristics of AKD were consistently shown across the differences in countries’ national income, reinforcing the generalizability of these findings. Future studies can utilize these findings to improve the assessment and management of patients with AKI based on phenotypic characterization of baseline health kidney health.

Key learning points:
What was known: Chronic kidney disease (CKD), defined as kidney dysfunction persisting more than 3 months, is associated with higher risk of incident acute kidney injury (AKI), death, and non-kidney recovery in patients with COVID-19. However, the influence of baseline acute kidney disease (AKD, kidney dysfunction with a duration of ≤3 months) on the courses and outcomes of COVID-19-associated AKI is unknown.
This study adds: In this multinational, multicenter, retrospective cohort study that included 4158 hospitalized adult COVID-19 patients with kidney disease, nearly two-thirds of non-end-stage kidney disease patients or kidney transplant recipients had underlying kidney dysfunction, with 55% identified as having baseline AKD. Similar to the AKI characteristics seen in CKD, community-acquired AKI was more frequent, and the risk of in-hospital death or non-kidney recovery was higher in AKD compared to patients with no kidney disease. These findings were consistent across national income categories.
Potential impact: The findings emphasize the need for the awareness of baseline kidney health status and targeted interventions in hospitalized COVID-19 patients.

## Figures and Tables

**Table 1:** Differences in patient characteristics and patient outcomes by kidney function.

	All (*n* = 4158)	AKI (*n* = 3038, 73.1%)	No AKI (*n* = 238, 5.7%)	ESKD (*n* = 882, 21.2%)
Demographics				
Age, years (median, IQR)	64 (52–74)	65 (53.7–77) *	66 (55–76) *	56 (45–66)
Age, years (mean ± SD)	62.7 ± 16.1	64.7 ± 15.7 *	64.9 ± 16.9 *	55.4 ± 15.2
Male, *n* (%)	2639 (63.5)	1926 (63.4)	146 (61.3)	567 (64.3)
Race, *n* (%)				
White	1051 (25.3)	918 (30.2) *	71 (29.8) *	62 (7.0)
Black	630 (15.2)	531 (17.5) *§	17 (7.1)	82 (9.3)
Hispanic or Latino	891 (21.4)	712 (23.4) *	42 (17.6)	137 (15.5)
Asian	1404 (33.8)	736 (24.2) *§	92 (38.7) *	576 (65.3)
Others	182 (4.4)	141 (4.6)	16 (6.7) *	25 (2.8)
Continent, *n* (%)				
North America	1697 (40.8)	1428 (47.0) *	112 (47.1) *	157 (17.8)
South America	765 (18.4)	607 (20.0) *§	31 (13.0)	127 (14.4)
Europe	368 (8.9)	336 (11.1) *§	8 (3.4)	24 (2.7)
Asia	1328 (31.9)	667 (22.0) *§	87 (36.6) *	574 (65.1)
National income				
HICs	2223 (53.5)	1887 (62.1) *	145 (60.9) *	191 (21.7)
UMICs	1194 (28.7)	812 (26.7) *§	33 (3.9) *	349 (39.6)
LMICs	741 (17.8)	339 (11.2) *§	60 (25.2) *	342 (38.8)
Weight, kg (median, IQR)	79.9 (65.3–96.9)	81.6 (68.0–98.9) *	77.0 (63.2–92.9) *	69.1 (58.0–81.0)
Height, m (median, IQR)	1.7 (1.6–1.8)	1.7 (1.6–1.8)	1.7 (1.6–1.8)	1.7 (1.6–1.7)
BMI				
Male, kg/m^2^ (median, IQR)	27.6 (23.8–32.1)	28.1 (24.2–32.7)	25.9 (23.1–29.6)	24.8 (22.4–28.1)
Female, kg/m^2^ (median, IQR)	28.3 (23.4–35.2)	29.0 (24.1–35.9)	27.0 (22.4–34.6)	25.4 (21.8–31.6)
Comorbidities, *n* (%)				
Hypertension	2530 (61.1)	2530 (61.1) *	168 (70.6)	674 (76.5)
Diabetes	1747 (42.4)	1747 (42.4) *	111 (46.6)	450 (51.1)
Cardiovascular disease	770 (18.7)	567 (18.9) §	61 (25.6) *	142 (16.2)
Chronic kidney disease (including KT)	1137 (27.4)	900 (29.6)	238 (100)	882 (100)
Lung disease	464 (11.3)	383 (12.8) *	32 (16.4) *	49 (5.6)
Congestive heart failure	335 (8.2)	266 (8.9) *	21 (8.8)	48 (5.5)
Active malignancy	257 (6.2)	221 (7.3) *	18 (7.6) *	18 (2.0)
Asthma	165 (4.0)	139 (4.6) *§	8 (3.4) *	18 (2.0)
Other immunodeficiency ^#^	181 (4.4)	141 (4.8) *§	20 (8.4) *	20 (2.3)
Liver disease	149 (3.6)	113 (3.8)	10 (4.2)	26 (3.0)
HIV infection	27 (0.7)	23 (0.8)	0 (0.0)	4 (0.5)
Total numbers of comorbidities (median, IQR)	2 (1–2)	1 (0–2) *§	2 (1–3)	2 (1–2)
Smoking (*n*, %)				
Non smoker	1767 (42.5)	1284 (46.9)	98 (45.0)	385 (47.4)
Former smoker	669 (17.7)	520 (19.0) *	46 (21.1) *	103 (12.7)
Current smoker	226 (6.0)	194 (7.1) *§	5 (2.3)	27 (3.3)
Unknown	1109 (29.4)	742 (27.1) *	69 (31.7)	298 (36.7)
Pre-hospitalization medication, *n* (%)				
Statin	1205 (29.8)	874 (29.9) §	92 (38.7)	239 (27.2) §
Beta blocker	889 (22.1)	667 (22.9) *	58 (24.4)	164 (18.7)
Diuretics	785 (19.5)	591 (20.3) *	53 (22.3)	141 (16.1)
ACEI	556 (13.9)	440 (15.2) *	43 (18.1) *	73 (8.4)
ARB	572 (14.2)	443 (15.3) *	36 (15.1)	93 (10.6)
Anticoagulant agent	463 (11.5)	368 (12.7) *	43 (18.1) *	52 (5.9)
Immunosuppressant	291 (7.2)	227 (7.8) *§	36 (15.1) *	28 (3.2)
NSAID	230 (5.7)	191 (6.6) *	21 (8.8) *	18 (2.1)
Herbal medication	19 (0.5)	17 (0.6)	0 (0.0)	2 (0.2)
Total numbers of medications (median, IQR)	2 (1–2)	1 (0–2) *§	2 (1–3) *	2 (1–2)
Enrollment at any kind of clinical trials				
Yes	356 (9.4)	285 (10.5) *§	41 (18.4) *	30 (3.6)
Reasons for hospital admission, *n* (%)				
Respiratory diagnosis	2525 (61.1)	1941 (63.4) *	141 (59.2)	470 (53.6)
Non-respiratory infection	1638 (39.8)	1300 (43.5) *§	68 (28.7)	270 (30.8)
Worsening renal dysfunction	972 (23.5)	656 (21.7) *§	30 (12.6)	286 (32.5) §
Metabolic issues ^##^	616 (15.0)	365 (12.2) *	41 (17.2)	210 (23.9)
Sepsis	640 (15.4)	534 (17.6) *§	17 (7.1)	89 (10.1)
Shock/hemodynamic instability	350 (8.5)	275 (9.1)	15 (6.3)	60 (6.8)
Heart diagnosis	304 (7.4)	255 (8.5) *	15 (6.3)	34 (3.9)
Central nervous system	120 (2.9)	97 (3.2)	5 (2.1)	18 (2.1)
Trauma	50 (1.2)	37 (1.2) §	8 (3.4)	5 (0.6) §
Postsurgical	35 (0.8)	33 (1.1) *	1 (0.4)	1 (0.1)
Others	579 (14.9)	437 (15.7) *	48 (20.3) *	94 (10.9)
Processes of care				
ICU care, yes (%)	1882 (45.3)	1593 (52.4) *§	37 (15.5) *	252 (28.6)
ECMO, yes (%)	174 (4.3)	158 (5.3) *§	1 (0.4)	15 (1.7)
Vent care, yes (%)	1587 (38.3)	1379 (45.6) *§	21 (8.8) *	187 (21.3)
Dialysis, yes (%)	1797 (43.4)	908 (30.1) *§	39 (16.4) *	850 (96.4)
Vent and dialysis, *n* (%)	960 (23.2)	766 (25.4) *§	8 (3.4) *	186 (21.1)
KRT modality among KRT received patients				
IHD only	1064 (59.4)	317 (35.1) *§	33 (84.6)	714 (84.0)
CKRT only	353 (19.7)	324 (35.9) *§	1 (2.6)	28 (3.3)
Both of IHD and CKRT	191 (10.7)	159 (17.6) *§	1 (2.6)	31 (3.6)
Others (UF or PD)	183 (10.2)	102 (11.3)	4 (10.3)	77 (9.1)
Enrolled at any kind of investigative trial, yes	356 (9.4)	285 (10.5) *§	41 (18.3) *	30 (3.6)
Patient outcome				
Length of stay, days (median, IQR)	12 (7–21)	13 (7–23) *§	8 (4–13)	11 (7–17.3)
Death, *n* (%)	1530 (38.4)	1249 (42.5) *§	34 (15.2) *	247 (30.0)
Among CKD patients without AKI				
CKD progression, *n* (%)	NA	NA	6 (3.1)	NA
Among survivors	*n* = 2453	*n* = 1688	*n* = 189	*n* = 576
Discharge information				
Home	1808 (73.8)	1155 (68.5) *§	157 (83.1)	496 (86.1)
Nursing home	290 (11.8)	234 (13.9) *	25 (13.2) *	31 (5.4)
Other healthcare facility	253 (10.3)	216 (12.8) *§	2 (1.1) *	35 (6.1)
Other	99 (4.0)	80 (4.7) *	5 (2.6)	14 (2.4)

* *p* < 0.05 compared to ESKD; § *p* < 0.05 compared to no AKI. Information on missing data is summarized in the Table S4. ^#^ Other immunodeficiency includes rheumatic disease, cyclic neutropenia, and any conditions that affect immune status. ^##^ Metabolic issues include metabolic acidosis, hyperkalemia, hyponatremia, or any other metabolic issues requiring hospital admission. Abbreviations: AKI, acute kidney injury; ESKD, end-stage kidney disease; IQR, inter quartile range; SD, standard deviation; HICs, high-income countries; UMICs, upper-middle-income countries; LMICs, lower-middle-income countries; BMI, body mass index; KT, kidney transplant recipients; HIV, human immunodeficiency virus; ACEI, angiotensin-converting enzyme inhibitor; ARB, angiotensin receptor blocker; NSAID, non-steroidal anti-inflammatory drug; ICU, intensive care unit; ECMO, extracorporeal membrane oxygenation; Vent, ventilator; KRT, kidney replacement therapy; IHD, intermittent hemodialysis; CKRT, continuous kidney replacement therapy; UF, ultrafiltration; PD, peritoneal dialysis; CKD, chronic kidney disease; NA, non-available.

**Table 2:** Differences in patient characteristics and outcomes in acute kidney injury patients by baseline kidney health status.

	NKD (*n* = 965, 31.8%)	AKD (*n* = 1173, 38.6%)	CKD (*n* = 799, 26.3%)	KT (*n* = 101, 3.3%)
Demographics				
Age, years (median, IQR)	61 (51–70) *§¶	68 (58–78) ¶	71 (59–80) ¶	53 (39–64.5)
Male, *n* (%)	686 (71.1) *§¶	687 (56.8)	499 (62.5)	54 (53.5)
Race, *n* (%)				
White	266 (27.6)	360 (30.7)	260 (32.5)	32 (31.7)
Black	86 (8.9) *¶	231 (19.7) ¶	174 (21.8) ¶	40 (39.6)
Hispanic or Latino	309 (32.0) *§¶	263 (22.4) ¶	125 (15.6)	15 (14.9)
Asian	251 (26.0) ¶	277 (23.6)	195 (24.4)	13 (12.9)
Others	53 (5.5)	42 (3.6)	45 (5.6)	1 (1.0)
Continent, *n* (%)				
North America	330 (34.2) *§¶	600 (51.2) ¶	424 (53.1) ¶	74 (73.3)
South America	261 (27.0) *§¶	233 (19.9) §¶	104 (13.0)	9 (8.9)
Europe	145 (15.0) *	87 (7.4) §	98 (12.3)	6 (5.9)
Asia	229 (23.7) ¶	253 (21.6)	173 (21.7)	12 (11.9)
National income				
HICs	539 (55.9) *§¶	725 (61.8) §¶	542 (67.8)	81 (80.2)
UMICs	355 (36.8) *§	295 (25.1)	151 (18.9)	11 (10.9)
LMICs	71 (7.4) *§¶	153 (13.0) ¶	106 (13.3) ¶	9 (8.9)
Weight, kg (median, IQR)	83.9 (60.0–100.0)	81.1 (66.0–98.9)	81.6 (68.0–97.9)	81.6 (65.0–98.0)
Height, m (median, IQR)	1.7 (1.6–1.8)	1.7 (1.6–1.8)	1.7 (1.6–1.8)	1.7 (1.6–1.8)
BMI				
Male, kg/m^2^ (median, IQR)	28.4 (25.3–32.7)	27.8 (23.9–32.9)	27.6 (23.7–32.3)	29.4 (25.0–32.9)
Female, kg/m^2^ (median, IQR)	28.3 (23.2–34.9)	28.3 (23.1–35.8)	30.9 (24.9–37.2)	29.4 (23.1–34.1)
Comorbidities, *n* (%)				
Hypertension	414 (43.6) *§	656 (56.0)	556 (69.6)	62 (61.4)
Diabetes	293 (31.1) *§	442 (38.0) §	409 (51.3)	42 (41.6)
Cardiovascular disease	120 (12.8) *§	206 (17.7) §	226 (28.4)	15 (14.9)
Lung disease	114 (12.1)	138 (11.9)	126 (15.9) ¶	5 (5.0)
Congestive heart failure	48 (5.2) §	79 (6.8) §	133 (16.8) ¶	6 (5.9)
Active malignancy	62 (6.5)	90 (7.7)	63 (7.9)	6 (5.9)
Asthma	59 (6.3)	49 (4.2)	30 (3.8)	1 (1.0)
Other immunodeficiency ^#^	46 (5.0) *¶	38 (3.3) ¶	45 (5.7) ¶	12 (11.9)
Liver disease	33 (3.5)	34 (2.9)	40 (5.0)	6 (5.9)
HIV infection	11 (1.2)	7 (0.6)	5 (0.6)	0
Total numbers of comorbidities (median, IQR)	1 (0–2) *§	1 (0–2) §	2 (1–3) ¶	2 (0–2)
Smoking (*n*, %)				
Non smoker	381 (45.1) ¶	491 (46.3)	355 (48.1)	57 (59.4)
Former smoker	139 (16.4)	210 (19.8)	146 (19.8)	25 (26.0)
Current smoker	71 (8.4)	80 (7.5)	42 (5.7)	1 (1.0)
Unknown	254 (30.1) ¶	280 (26.4) ¶	195 (26.4) ¶	13 (13.5)
Pre-hospitalization medication, *n* (%)				
Statin	196 (21.5) *§	316 (27.9) §	324 (41.4)	38 (38.0)
Beta blocker	132 (14.6) *§	232 (20.5) §	257 (32.8)	46 (46.0)
Diuretics	99 (10.9) *§	214 (19.0)	249 (31.8)	29 (29.0)
ACEI	132 (14.6) ¶	153 (13.7)	150 (19.3) ¶	5 (5.0)
ARB	113 (12.5) *	193 (17.2)	123 (15.8)	14 (14.0)
Anticoagulant agent	77 (8.6) *§	142 (12.6)	139 (17.9)	10 (10.0)
Immunosuppressant	56 (6.2) ¶	50 (4.4) ¶	57 (7.3) ¶	64 (63.4)
NSAID	62 (6.9)	60 (5.3)	63 (8.1)	6 (6.0)
Herbal medication	5 (0.6)	8 (0.7)	3 (0.4)	1 (0.1)
Total numbers of medications (median, IQR)	0 (0–1) *§¶	1 (0–2) §¶	2 (0–3) ¶	2 (0–3)
Reasons for hospital admission, *n* (%)				
Respiratory diagnosis	675 (71.0) *§¶	721 (61.6)	469 (58.8)	49 (48.5)
Non-respiratory infection	454 (48.3) *§	484 (41.8)	325 (41.2)	37 (36.6)
Worsening renal dysfunction	42 (4.4) *§¶	337 (28.9)	239 (30.0)	38 (37.6)
Metabolic issues ^##^	76 (8.2) *§	163 (14.1)	114 (14.3)	12 (11.9)
Sepsis	172 (17.9)	229 (19.6)	123 (15.4)	10 (9.9)
Shock/hemodynamic instability	86 (9.1) ¶	128 (11.0) ¶	60 (7.5)	1 (1.0)
Heart diagnosis	39 (4.2) *§	113 (9.7)	96 (12.1)	7 (6.9)
Central nervous system	21 (2.2) §	39 (3.4)	36 (4.5)	1 (1.0)
Trauma	13 (1.4)	13 (1.1)	11 (1.4)	0
Postsurgical	15 (1.6)	13 (1.1)	4 (0.5)	1 (1.0)
Others	73 (8.7) *§¶	203 (18.5)	141 (18.8)	20 (20.2)
AKI diagnosis criteria				
Oliguria	23 (3.6) *¶	76 (9.2) ¶	40 (6.9)	9 (12.0)
Increasing serum creatinine criteria	485 (76.0) *§¶	423 (51.2)	328 (56.8)	38 (50.7)
Decreasing serum creatinine criteria	130 (20.4) *§¶	327 (39.6)	209 (36.2)	28 (37.3)
AKI development time				
Community acquired (<48 h)	369 (43.3) *§	918 (83.0) §	586 (77.7)	81 (81.0)
Hospital acquired (≥48 h)	484 (56.7) *§	188 (17.0) §	168 (22.3)	19 (19.0)
Volume status at admission, *n* (%)				
Euvolemic	509 (52.7) *§	515 (43.9)	323 (40.4) *¶	55 (54.5)
Dehydrated	170 (17.6) *§¶	400 (34.1)	242 (30.3)	33 (32.7)
Overloaded	82 (8.5) §	114 (9.7) §	113 (14.1)	9 (8.9)
AKI severity at diagnosis				
SCr at diagnosis, mg/dL, median (IQR)	1.4 (1.1–1.9) §	1.9 (1.5–2.7)	2.5 (1.8–4.2)	2.5 (1.8–4.7)
Stage 1, %	492 (64.7) *§	770 (74.3)	526 (74.0)	65 (66.3)
Stage 2, %	104 (13.7) *§	86 (8.3)	61 (8.6)	12 (12.2)
Stage 3, %	55 (7.2)	58 (5.6)	30 (4.2)	7 (7.1)
Stage 3D, %	110 (14.5)	123 (11.9)	94 (13.2)	14 (14.3)
AKI severity at peak				
Peak SCr, mg/dL, median (IQR)	1.9 (1.3–3.8) *§¶	2.4 (1.6–4.0) §	3.2 (2.0–5.0)	2.8 (1.9–5.2)
Stage 1, %	356 (40.9) *§	585 (53.6)	416 (54.2)	48 (48.0)
Stage 2, %	111 (12.7)	111 (10.2)	79 (10.3)	14 (14.0)
Stage 3, %	86 (9.9) §	75 (6.9)	34 (4.4)	4 (4.0)
Stage 3D, %	315 (36.2) *	320 (29.3)	239 (31.1)	34 (34.0)
Processes of care				
ICU care, yes (%)	642 (66.5) *§	593 (50.6) §	318 (39.8)	40 (39.2)
ECMO, yes (%)	68 (7.2) §	65 (5.6) §	23 (2.9)	2 (2.0)
Vent care, yes (%)	626 (65.3)*§	474 (40.4) §¶	246 (30.9)	33 (32.7)
Dialysis, yes (%)	315 (32.9) *	320 (27.4)	239 (30.1)	34 (33.7)
Vent and dialysis, *n* (%)	313 (32.7) *	261 (22.3)	169 (21.3)	23 (22.8)
KRT modality among KRT received patients				
IHD only	78 (24.8) *§	116 (36.4)	110 (46.8)	13 (39.4)
CKRT only	151 (47.9) *§	97 (30.4)	67 (28.5)	9 (27.3)
Both of IHD and CKRT	57 (18.1)	61 (19.1)	36 (15.3)	5 (15.2)
Others = (UF or PD)	29 (9.2)	45 (14.1)	22 (9.4)	6 (18.2)
Enrolled at any kind of investigative trial, yes	108 (12.7) *	85 (8.1)	81 (11.2)	11 (12.1)
Patient outcome				
Length of stay, days (median, IQR)	17 (9–30) *§	11 (6–21)	11 (6–19)	12 (7–19)
Death	434 (47.1) §¶	481 (42.4) §¶	310 (39.7)	24 (24.0)
Combined outcome	448 (48.6)	516 (45.5)	350 (44.9)	39 (39.0)
Among survivors	*n* = 488	*n* = 654	*n* = 470	*n* = 76
Kidney outcome				
AKI recovery				
Complete, %	349 (71.5) §¶	449 (68.5) §¶	178 (37.9)	32 (42.1)
Partial, %	116 (23.8) §¶	160 (24.5) §¶	247 (52.6)	28 (36.8)
Non-recovery, %	14 (2.9) §¶	35 (5.4) ¶	40 (8.5) ¶	15 (19.7)
Discharge information				
Home	336 (68.9) ¶	431 (66.1) ¶	323 (68.9) ¶	65 (85.5)
Nursing home	48 (9.8)* §	102 (15.6)	77 (16.4)	7 (9.2)
Other healthcare facility	78 (16.0) ¶	87 (13.3) ¶	49 (10.4)	2 (2.6)
Other	26 (5.3)	32 (4.9)	20 (4.3)	2 (2.6)

* p < 0.05 compared to AKD; § *p* < 0.05 compared to CKD; ¶ *p* < 0.05 compared to KT. Note: Information on missing data is summarized in the Table S4. ^#^ Other immunodeficiency includes rheumatic disease, cyclic neutropenia, and any conditions that affect immune status. ^##^ Metabolic issues include metabolic acidosis, hyperkalemia, hyponatremia, or any other metabolic issues requiring hospital admission. Abbreviations: NKD, no kidney disease; AKD, acute kidney disease; CKD, chronic kidney disease; KT, kidney recipients; IQR, inter quartile range; *n*, number; HICs, high-income countries; UMICs, upper-middle-income countries; LMICs, lower-middle-income countries; BMI, body mass index; HIV, human immunodeficiency virus; ACEI, angiotensin-converting enzyme inhibitor; ARB, angiotensin receptor blocker; NSAID, non-steroidal anti-inflammatory drug; AKI, acute kidney injury; SCr, serum creatinine; ICU, intensive care unit; ECMO, extracorporeal membrane oxygenation; Vent, ventilator; KRT, kidney replacement therapy; IHD, intermittent hemodialysis; CKRT, continuous kidney replacement therapy; UF, ultrafiltration; PD, peritoneal dialysis.

**Table 3:** Patient and renal outcome at hospital discharge.

	Event Rate (%)	Crude OR	Age, Sex-Matched OR	Multivariable Matched OR
In-hospital mortality (*n* = 4158)				
ESKD	247 (30.0)	Reference	Reference	Reference
No AKI	34 (15.2)	0.407 (0.273, 0.607)	0.351 (0.235, 0.524)	0.531 (0.316, 0.893)
Any AKI	1249 (42.5)	1.726 (1.462, 2.037)	1.452 (1.222, 1.724)	1.099 (0.795, 1.519)
In-hospital mortality among AKI patients (*n* = 2937)				
NKD	434 (47.1)	Reference	Reference	Reference
AKD	481 (42.4)	0.827 (0.694, 0.985)	0.720 (0.600, 0.865)	1.244 (0.953, 1.623)
CKD	310 (39.7)	0.742 (0.611, 0.900)	0.613 (0.500, 0.750)	1.270 (0.939, 1.717)
KT	24 (24.0)	0.355 (0.220, 0.572)	0.416 (0.256, 0.674)	0.878 (0.471, 1.637)
Combined outcome among AKI patients (*n* = 2937)				
NKD	448 (48.5)	Reference	Reference	Reference
AKD	516 (45.5)	0.873 (0.728, 1.046)	0.797 (0.665, 0.954)	1.502 (1.0736, 2.102)
CKD	350 (44.9)	0.785 (0.642, 0.960)	0.750 (0.615, 0.915)	1.604 (1.108, 2.322)
KT	39 (39.0)	0.744 (0.485, 1.141)	0.781 (0.509, 1.199)	2.144 (1.116, 4.117)
Partial or non-renal recovery at hospital discharge among AKI survivors (*n* = 1688)				
NKD	139 (28.5)	Reference	Reference	Reference
AKD	205 (31.3)	1.146 (0.887, 1.482)	1.197 (0.917, 1.561)	1.286 (0.921, 1.795)
CKD	292 (62.1)	4.119 (3.141, 5.400)	4.412 (3.328, 5.850)	5.519 (3.912, 7.786)
KT	44 (57.9)	3.452 (2.102, 5.669)	3.454 (2.092, 5.705)	4.044 (2.291, 7.139)

Abbreviations: OR, odds ratio; AKI, acute kidney injury; NKD, no kidney disease; AKD, acute kidney disease; CKD, chronic kidney disease; KT, kidney transplant; ESKD, end-stage kidney disease. Note: Multivariable model for in-hospital mortality was adjusted with age, race, hypertension, congestive heart failure, liver disease, national income, statin, investigational drug, shock at admission, ICU use, processes of care, and kidney health status. Multivariable model for in-hospital mortality among AKI survivors was adjusted with age, race, national income status, congestive heart failure, liver disease, lung disease, statin, investigational drug, AKI stage, process of care, kidney recovery, and baseline kidney function status. Multivariable model for combined outcome among AKI survivors was adjusted with age, race, congestive heart failure, liver disease, national income, statin, investigational drug, AKI development time, AKI stage at peak, processes of care, and baseline kidney function status. Multivariable model for partial or non-recovery among survivors was adjusted with race, national income, AKI development time, AKI stage at peak, and baseline kidney function status.

## Data Availability

The datasets used and/or analyzed during the current study are available from the corresponding author on reasonable request.

## Supplementary Materials

The following supporting information can be downloaded at https://www.mdpi.com/article/10.3390/jcm14041212/s1. Table S1: List of sites and COVID-19 Global Snapshot investigators; Table S2: Electronic case report forms; Table S3: Definitions; Table S4: Number of missing information for collected data; Table S5: AKI characteristics and outcomes, stratified by national income status; Table S6: Multivariable binary regression model predicting in-hospital mortality in all patients; Table S7: Multivariable binary regression models predicting in-hospital mortality and combined outcome in AKI patients; Table S8: In-hospital mortality by national income among AKI patients; Table S9: Factors associated with partial or non-renal recovery among AKI survivors.
