# Supplementary material for: Influence of Baseline Kidney Function on Patient and Kidney Outcomes in Patients with COVID-19: A Multi-National Observational Study
Source: J Clin Med. 2025 Feb 12;14(4):1212. doi: 10.3390/jcm14041212 (PMC11856477; doi:10.3390/jcm14041212)
Supplement: Supplementary file 1 [file jcm-14-01212-s001.zip › Supplement material 2_eCRF.pdf]

COVID-19 Infection in Patients with Kidney Disease: Patient Data Enrollment - Page 1

Patient Demographics:

Patient reference ID: | Gender: | Age: | Race:

Weight (kg): | Weight (lb): | Height (cm): | Height (inch): | BMI:

Site Code: | Subject ID: | Form Version:

Which section do you want to work on:

Enrollment Date: | Location where patient was seen:

COVID-19 antigen status: | Test positive date: | Source of COVID-19 infection:

Is patient on any investigative trial?

Signs/Symptoms: (check all that apply)

- ☐ Cough ☐ Sputum ☐ Dyspnea ☐ Fever
- ☐ Myalgia ☐ Vomiting ☐ Nausea ☐ Diarrhea
- ☐ Headache ☐ Hemoptysis ☐ Confusion ☐ Loss of smell/taste

Comorbidities/Prior Medical Conditions: (mark all that apply)

- ☐ Liver ☐ Lung ☐ CVD ☐ CHF
- ☐ DM ☐ HTN ☐ HIV ☐ Active malignancy
- ☐ Asthma ☐ Other immunodeficiency

Smoking status:

Home Medication: (check all that apply)

- ☐ NSAID ☐ ARB ☐ ACE-I ☐ Immunosuppressant
- ☐ Beta-Blocker ☐ Statin ☐ Diuretic ☐ Anticoagulant agent
- ☐ Herbal medication

Please continue with patient enrollment data on next page.

Enrollment - Page 2

Does patient have a known history of chronic kidney disease?

Does patient have a functioning kidney transplant?

Is patient with ESKD (chronic dialysis dependent)?

Baseline Creatinine - Latest measured SCr in the last 3 - 12 months prior to this hospitalization

Baseline SCr available?

Date obtained:

Creatinine (mg/dL):

Creatinine (umol/L):

CKD-EPI eGFR:

Hospital Admission Creatinine - First creatinine available during this admission

Admission SCr available?

Date obtained:

Creatinine (mg/dL):

Creatinine (umol/L):

CKD-EPI eGFR:

Reasons for hospital/ICU admission: (check all that apply)

- ☐ Shock/Hemodynamic instability
- ☐ Sepsis
- ☐ Infection
- ☐ Heart diagnosis
- ☐ Respiratory diagnosis
- ☐ Post-surgical
- ☐ Central nervous system
- ☐ Trauma
- ☐ Metabolic issues
- ☐ Worsening renal function
- ☐ Other (please specify)

Other reason for admission:

Please continue with patient enrollment data on next page.

Enrollment - Page 3

Did patient meet AKI criteria? [ ]

Which of the following AKI criteria did patient meet? (check all that apply)

- ☐ An abrupt (within 48 hrs) increase in SCr >= 0.3 mg/dL (26.5 umol/L)
- ☐ An abrupt increase in SCr >= 50% from reference
- ☐ Decrease of SCr >= 0.3 mg/dL (26.5 umol/L) in 48 hrs
- ☐ Decrease of SCr >= 50% within 7 days
- ☐ Documented oliguria of less than 0.3 ml/kg/hr for 6 hours or longer

AKI diagnosis date: [ ] SCr (mg/dL) on AKI date: [ ] SCr (umol/L) on AKI date: [ ]

Please answer following questions for enrollment day:

Patient fluid status: [ ]

Has the patient been oliguric (UO < 500 ml/24hr)? [ ]

Has patient received vasopressors? [ ]

Has patient received diuretics? [ ]

Has patient received invasive mechanical ventilation? [ ]

Has patient received ECMO? [ ]

O2 requirement: [ ]

Has patient been placed in prone position? [ ]

Received dialysis? [ ]

First Dialysis:

Date of first dialysis: [ ] SCr (mg/dL) at dialysis start: [ ] SCr (umol/L) at dialysis start: [ ]

Main reasons for dialysis initiation: (check all that apply)

☐ Fluid overload ☐ Uremia ☐ Anuria ☐ Metabolic acidosis
☐ Respiratory failure ☐ Cytokine storm ☐ Electrolyte abnormalities

Mark all types of dialysis patient received:

☐ IHD ☐ UF ☐ PD ☐ CVVH
☐ CVVHD ☐ CVVHDF ☐ SHVHF ☐ SLED

NOTE: You have reached the end of Enrollment section. Please make sure all required fields are properly answered and remember to click Submit or Update command, or File icon to save the patient enrollment data. Do not proceed to outcome assessment pages until appropriate due time.

Go to top

Outcome Info - Assessment 1
Patient status from enrollment to current assessment day

Assessment 1 date: ____ Patient location: [▾]

Patient fluid status:	[▾]
Has patient been oliguric (UO < 500 ml/24hr)?	[▾]
Is patient still oliguric (UO < 500 ml/24hr)?	[▾]
Has patient received vasopressors?	[▾]
Is patient still on vasopressors?	[▾]
Has patient received diuretics?	[▾]
Is patient still on diuretics?	[▾]
Has patient received invasive mechanical ventilation?	[▾]
Is patient still on invasive mechanial ventilation?	[▾]
Has patient received ECMO?	[▾]
Is patient still on ECMO?	[▾]
O2 requirement:	[▾]
Has patient been placed in prone position?	[▾]
Received dialysis?	[▾]
Is patient still on dialysis?	[▾]

First Dialysis:

Date of first dialysis: ____ SCr (mg/dL) at dialysis start: ____ SCr (umol/L) at dialysis start: ____

Main reasons for dialysis initiation/continuation: (check all that apply)

- ☐ Fluid overload
- ☐ Uremia
- ☐ Anuria
- ☐ Metabolic acidosis
- ☐ Respiratory failure
- ☐ Cytokine storm
- ☐ Electrolyte abnormalities

Mark all types of dialysis patient received:

- ☐ IHD
- ☐ UF
- ☐ PD
- ☐ CVVH
- ☐ CVVHD
- ☐ CVVHDF
- ☐ SHVHF
- ☐ SLED

Last Available Creatinine:

Date of SCr: ____ Last SCr (mg/dL): ____ Last SCr (umol/L): ____

Is the patient discharged? [▾]

NOTE: You have reached the end of Outcome Assessment 1. Please make sure all required fields are properly answered and rember to click Update command or File icon to save the page. Do not proceed to other outcome assessments until appropriate due time. If the patient has been discharged, please continue to fill out the discharge information on the last page of this form.

Go to top

Outcome Info - Assessment 2

Patient status from last assessment to current assessment day

Assessment 2 date: ____ Patient location: ____

Patient fluid status: ____

Has patient been oliguric (UO < 500 ml/24hr)? ____

Is patient still oliguric (UO < 500 ml/24hr)? ____

Has patient received vasopressors? ____

Is patient still on vasopressors? ____

Has patient received diuretics? ____

Is patient still on diuretics? ____

Has patient received invasive mechanical ventilation? ____

Is patient still on invasive mechanial ventilation? ____

Has patient received ECMO? ____

Is patient still on ECMO? ____

O2 requirement: ____

Has patient been placed in prone position? ____

Received dialysis? ____

Is patient still on dialysis? ____

First Dialysis:

Date of first dialysis: ____ SCr (mg/dL) at dialysis satrt: ____ SCr (umol/L) at dialysis start: ____

Main reasons for dialysis initiation/continuation: (check all that apply)

- ☐ Fluid overload
- ☐ Uremia
- ☐ Anuria
- ☐ Metabolic acidosis
- ☐ Respiratory failure
- ☐ Cytokine storm
- ☐ Electrolyte abnormalities

Mark all types of dialysis patient received:

- ☐ IHD
- ☐ UF
- ☐ PD
- ☐ CVVH
- ☐ CVVHD
- ☐ CVVHDF
- ☐ SHVHF
- ☐ SLED

Last Available Creatinine:

Date of SCr: ____ Last SCr (mg/dL): ____ Last SCr (umol/L): ____

Is the patient discharged? ____

NOTE: You have reached the end of Outcome Assessment 2. Please make sure all required fields are properly answered and rember to click Update command or File icon to save the page. Do not proceed to other outcome assessments until appropriate due time. If the patient has been discharged, please continue to fill out the discharge information on the last page of this form.

Go to top

Outcome Info - Assessment 3

Patient status from enrollment to current assessment day

Assessment 3 date: [ ]
Patient location: [▾]

Patient fluid status:	[▾]
Has patient been oliguric (UO < 500 ml/24hr)?	[▾]
Is patient still oliguric (UO < 500 ml/24hr)?	[▾]
Has patient received vasopressors?	[▾]
Is patient still on vasopressors?	[▾]
Has patient received diuretics?	[▾]
Is patient still on diuretics?	[▾]
Has patient received invasive mechanical ventilation?	[▾]
Is patient still on invasive mechanial ventilation?	[▾]
Has patient received ECMO?	[▾]
Is patient still on ECMO?	[▾]
O2 requirement:	[▾]
Has patient been placed in prone position?	[▾]
Received dialysis?	[▾]
Is patient still on dialysis?	[▾]

First Dialysis:

Date of first dialysis: [ ]
SCr (mg/dL) at dialysis start: [ ]
SCr (umol/L) at dialysis start: [ ]

Main reasons for dialysis initiation/continuation: (check all that apply)

- ☐ Fluid overload
- ☐ Uremia
- ☐ Anuria
- ☐ Metabolic acidosis
- ☐ Respiratory failure
- ☐ Cytokine storm
- ☐ Electrolyte abnormalities

Mark all types of dialysis patient received:

- ☐ IHD
- ☐ UF
- ☐ PD
- ☐ CVVH
- ☐ CVVHD
- ☐ CVVHDF
- ☐ SHVHF
- ☐ SLED

Last Available Creatinine:

Date of SCr: [ ]
Last SCr (mg/dL): [ ]
Last SCr (umol/L): [ ]

Is the patient discharged? [▾]

NOTE: You have reached the end of Outcome Assessment 3. Please make sure all required fields are properly answered and rember to click Update command or File icon to save the page. Do not proceed to other outcome assessments until appropriate due time. If the patient has been discharged, please continue to fill out the discharge information on the last page of this form.

Go to top

Outcome Info - Assessment 4
Patient status from enrollment to current assessment day

Assessment 4 date: ________ Patient location: [dropdown]

Patient fluid status: [dropdown]

Has patient been oliguric (UO < 500 ml/24hr)? [dropdown]

Is patient still oliguric (UO < 500 ml/24hr)? [dropdown]

Has patient received vasopressors? [dropdown]

Is patient still on vasopressors? [dropdown]

Has patient received diuretics? [dropdown]

Is patient still on diuretics? [dropdown]

Has patient received invasive mechanical ventilation? [dropdown]

Is patient still on invasive mechanial ventilation? [dropdown]

Has patient received ECMO? [dropdown]

Is patient still on ECMO? [dropdown]

O2 requirement: [dropdown]

Has patient been placed in prone position? [dropdown]

Received dialysis? [dropdown]

Is patient still on dialysis? [dropdown]

First Dialysis:

Date of first dialysis: ________ SCr (mg/dL) at dialysis start: ________ SCr (umol/L) at dialysis start: ________

Main reasons for dialysis initiation/continuation: (check all that apply)

- ☐ Fluid overload
- ☐ Uremia
- ☐ Anuria
- ☐ Metabolic acidosis
- ☐ Respiratory failure
- ☐ Cytokine storm
- ☐ Electrolyte abnormalities

Mark all types of dialysis patient received:

- ☐ IHD
- ☐ UF
- ☐ PD
- ☐ CVVH
- ☐ CVVHD
- ☐ CVVHDF
- ☐ SHVHF
- ☐ SLED

Last Available Creatinine:

Date of SCr: ________ Last SCr (mg/dL): ________ Last SCr (umol/L): ________

Is the patient discharged? [dropdown]

NOTE: You have reached the end of Outcome Assessment 4. Please make sure all required fields are properly answered and rember to click Update command or File icon to save the page. This is the last outcome assessment. If the patient has been discharged, please continue to fill out the discharge information on the last page of this form.

Go to top

Discharge Info
Patient status upon final discharge from hospital

Discharge date:

Discharge from:

Discharge to:

Did patient develop AKI?

SCr at discharge (mg/dL):

SCr at discharge (umol/L):

Date of peak SCr during hospital stay:

Peak SCr during hospital stay (mg/dL):

Peak SCr during hospital stay (umol/L):

Alive at discharge?

Kidney recovery at discharge:

Date of last dialysis:

COVID-19 antibody test performed?

COVID-19 antibody test date:

COVID-19 antibody test result:

COVID-19 antibody IgM:

COVID-19 antibody IgG:

Patient Died (please complete this section if patient has died at discharge)

Death location:

Please specify if other location of death:

Cause of death:

Please specify if other cause of death:

Was autopsy performed?

NOTE: You have reached the last section of data collection for this COVID-19 hospital admission. Please make sure all required fields are properly answered and remember to click Update command or File icon to save the page. Thank you for your participation.

Go to top

3-Month Follow-Up - Page 1

Patient status for the 3 months period after discharge from COVID-19 hospital admission

3-Month followup date:

How is data acquired?

Who supplies the information?

Is patient alive?

Please answer the following questions if patient is alive. Otherwise skip to the section titled "Patient Died".

Patient location:

Please specify if other health facility:

Is patient receiving any dialytic therapy?

Please specify type of dialysis patient receives:

Lab test done at this follow-up

Serum creatinine done?

Date of SCr lab:

SCr (mg/dL):

SCr (umol/L):

BUN done?

Date of BUN lab:

BUN (mg/dL):

BUN (mmol/L):

Urine protein done?

Date of urine protein lab:

Urine protein result:

COVID-19 antigen done?

COVID-19 antigen date:

COVID-19 antigen result:

COVID-19 antibody done?

COVID-19 antibody date:

COVID-19 antibody result:

Kidney recovery status:

New comorbidities since initial COVID-19 hospital admission (check all that apply)

- ☐ Liver
- ☐ Lung
- ☐ CVD
- ☐ CHF
- ☐ HTN
- ☐ DM
- ☐ CKD
- ☐ HIV
- ☐ Active malignancy
- ☐ Asthma
- ☐ Other immunodeficiency

Smoking status:

Home medications: (check all that apply)

- ☐ NSAID
- ☐ ARB
- ☐ ACE-I
- ☐ Immunosuppressant
- ☐ Beta-Blocker
- ☐ Statin
- ☐ Diuretic
- ☐ Anticoagulant agent
- ☐ Herbal medication

Readmissions since discharge from COVID-19 hospital admission

Readmitted to ED?

How many ED readmissions?

Readmitted to hospital?

How many hospital readmissions?

Total number of days in hospital:

Please continue with 3-month follow-up data on next page

3-Month Follow-Up - Page 2

EQ-5D-5L Health Questionnaire (mark descriptions which best describe today's health)

Mobility:

Self-Care

Usual Activities:

Pain/Discomfort:

Anxiety/Depression:

Rate today's health between 0 (worst) and 100 (best):

SF-8 Health Survey (your views about your health during the past 4 weeks)

Overall, how would you rate your health during the past 4 weeks?

How much did physical health problems limit your physical activities?

How much difficulty did you have doing your daily work because of your physical health?

How much bodily pain have you had?

How much energy did you have?

How much did physical health or emotional problems limit your usual social activities with family or friends?

How much have you been bothered by emotional problems (such as feeling anxious, depressed or irritable)?

How much did personal or emotional problems keep you from doing your usual work, school or other daily activities?

Patient Died

Death location:

Please specify if other location of death:

Cause of death:

Please specify if other cause of death:

Date of death:

Was autopsy performed?

Go to top

6-Month Follow-Up - Page 1

Patient status for the 3 months period since last assessment

6-Month followup date: [ ]

How is data acquired? [▾]

Who supplies the information? [▾]

Is patient alive? [▾]

Please answer the following questions if patient is alive. Otherwise skip to the section titled "Patient Died".

Patient location: [▾]

Please specify if other health facility: [ ]

Is patient receiving any dialytic therapy? [▾]

Please specify type of dialysis patient receives: [▾]

Lab test done at this follow-up

Serum creatinine done? [▾]	Date of SCr lab: [ ]	SCr (mg/dL): [ ]	SCr (umol/L): [ ]
BUN done? [▾]	Date of BUN lab: [ ]	BUN (mg/dL): [ ]	BUN (mmol/L): [ ]
Urine protein done? [▾]	Date of urine protein lab: [ ]	Urine protein result: [▾]	
COVID-19 antigen done? [▾]	COVID-19 antigen date: [ ]	COVID-19 antigen result: [▾]	
COVID-19 antibody done? [▾]	COVID-19 antibody date: [ ]	COVID-19 antibody result: [▾]	

Kidney recovery status: [▾]

New comorbidities since initial COVID-19 hospital admission (check all that apply)

- ☐ Liver ☐ Lung ☐ CVD ☐ CHF ☐ HTN
- ☐ DM ☐ CKD ☐ HIV ☐ Active malignancy
- ☐ Asthma ☐ Other immunodeficiency

Smoking status: [▾]

Home medications: (check all that apply)

- ☐ NSAID ☐ ARB ☐ ACE-I ☐ Immunosuppressant
- ☐ Beta-Blocker ☐ Statin ☐ Diuretic ☐ Anticoagulant agent
- ☐ Herbal medication

Readmissions since discharge from COVID-19 hospital admission

Readmitted to ED? [▾]

How many ED readmissions? [ ]

Readmitted to hospital? [▾]

How many hospital readmissions? [ ]

Total number of days in hospital: [ ]

Please continue with 6-month follow-up data on next page.

6-Month Follow-Up - Page 2

EQ-5D-5L Health Questionnaire (mark descriptions which best describe today's health)

Mobility:

Self-Care

Usual Activities:

Pain/Discomfort:

Anxiety/Depression:

Rate today's health between 0 (worst) and 100 (best):

SF-8 Health Survey (your views about your health during the past 4 weeks)

Overall, how would you rate your health during the past 4 weeks?

How much did physical health problems limit your physical activities?

How much difficulty did you have doing your daily work because of your physical health?

How much bodily pain have you had?

How much energy did you have?

How much did physical health or emotional problems limit your usual social activities with family or friends?

How much have you been bothered by emotional problems (such as feeling anxious, depressed or irritable)?

How much did personal or emotional problems keep you from doing your usual work, school or other daily activities?

Patient Died

Death location:

Please specify if other location of death:

Cause of death:

Please specify if other cause of death:

Date of death:

Was autopsy performed?

Go to top

12-Month Follow-Up - Page 1

Patient status for the 6 months period since last assessment

12-Month followup date:

How is data acquired?

Who supplies the information?

Is patient alive?

Please answer the following questions if patient is alive. Otherwise skip to the section titled "Patient Died".

Patient location:

Please specify if other health facility:

Is patient receiving any dialytic therapy?

Please specify type of dialysis patient receives:

Lab test done at this follow-up

Serum creatinine done?

Date of SCr lab:

SCr (mg/dL):

SCr (umol/L):

BUN done?

Date of BUN lab:

BUN (mg/dL):

BUN (mmol/L):

Urine protein done?

Date of urine protein lab:

Urine protein result:

COVID-19 antigen done?

COVID-19 antigen date:

COVID-19 antigen result:

COVID-19 antibody done?

COVID-19 antibody date:

COVID-19 antibody result:

Kidney recovery status:

New comorbidities since initial COVID-19 hospital admission (check all that apply)

- ☐ Liver
- ☐ Lung
- ☐ CVD
- ☐ CHF
- ☐ HTN
- ☐ DM
- ☐ CKD
- ☐ HIV
- ☐ Active malignancy
- ☐ Asthma
- ☐ Other immunodeficiency

Smoking status:

Home medications: (check all that apply)

- ☐ NSAID
- ☐ ARB
- ☐ ACE-I
- ☐ Immunosuppressant
- ☐ Beta-Blocker
- ☐ Statin
- ☐ Diuretic
- ☐ Anticoagulant agent
- ☐ Herbal medication

Readmissions since discharge from COVID-19 hospital admission

Readmitted to ED?

How many ED readmissions?

Readmitted to hospital?

How many hospital readmissions?

Total number of days in hospital:

Please continue with 12-month follow-up data on next page.

12-Month Follow-Up - Page

EQ-5D-5L Health Questionnaire (mark descriptions which best describe today's health)

Mobility:

Self-Care

Usual Activities:

Pain/Discomfort:

Anxiety/Depression:

Rate today's health between 0 (worst) and 100 (best):

SF-8 Health Survey (your views about your health during the past 4 weeks)

Overall, how would you rate your health during the past 4 weeks?

How much did physical health problems limit your physical activities?

How much difficulty did you have doing your daily work because of your physical health?

How much bodily pain have you had?

How much energy did you have?

How much did physical health or emotional problems limit your usual social activities with family or friends?

How much have you been bothered by emotional problems (such as feeling anxious, depressed or irritable)?

How much did personal or emotional problems keep you from doing your usual work, school or other daily activities?

Patient Died

Death location:

Please specify if other location of death:

Cause of death:

Please specify if other cause of death:

Date of death:

Was autopsy performed?

Go to top
