# Supplementary material for: Influence of Baseline Kidney Function on Patient and Kidney Outcomes in Patients with COVID-19: A Multi-National Observational Study
Source: J Clin Med. 2025 Feb 12;14(4):1212. doi: 10.3390/jcm14041212 (PMC11856477; doi:10.3390/jcm14041212)
Supplement: Supplementary file 1 [file jcm-14-01212-s001.zip › Supplement tables1.pdf]

Influence of baseline kidney function on patient and kidney outcomes in patients with COVID-19 : A multi-national observational study

COVID-19 Global Snapshot Study

Supplement Materials

COVID-19 Global Snapshot Investigators

eTable 1. List of sites and COVID-19 Global Snapshot investigators

GNI	Country	Site	Principle Investigator	Co-investigators
HICs	Canada	Sacré-Coeur de Montreal Hospital	Josée Bouchard	Anatolie Duca
	USA	University of California, San Diego	Ravindra L. Mehta	Harin Rhee<br>Etienne Macedo
		University of Colorado Anschutz Medical Center	Amber Suzanne Podoll	Anip Bansal,
		University Of Utah Medical Center	Josephine Abraham	Carpio, Louis
		University of Alabama	Eric Kimbell Judd	Vermeiren, Daisy<br>Samuel T Johnston
		University of Kentucky	Javier A. Neyra	Samuel C Beavin
		Mayo clinic	Kianoush B. Kashani	
		University of Virginia	Negiin Pourafsha	
		UPMC Mercy	John Kellum	
		St Peter's Healthcare Partners	Jorge Cerda	Deidra Knauth,<br>Abigail Mosenthin
		Sibley Memorial Hospital	Samir Gautam	Jonathan Lim
		UPMC East	Murugan Raghavan	Bollam, Rahul
		UPMC Magee Women’s Hospital	Murugan Raghavan	Bollam, Rahul
		UPMC McKeesport	Murugan Raghavan	Bollam, Rahul
		UPMC Presbyterian	Murugan Raghavan	Bollam, Rahul
		UPMC Shadyside	Murugan Raghavan	Bollam, Rahul
		UPMC St. Margaret's	Murugan Raghavan	Bollam, Rahul
	Chile	CLINICA DAVILA	Andrés D. Boltansky	Carlos E. Irarrázabal
	Austria	Medical University Innsbruck	Michael Joannidis	
	Ireland	Mater Misericordiae University Hospital	Patrick Murray	
		St. Vincent’s University Hospital	Patrick Murray	
	Italy	University of Piemonte Orientale	Vincenzo Cantaluppi	Umberto M. Morosini
	Netherlands	Radboud University Medical Center Nijmegen	Peter Pickkers	

	Spain	Hospital Universitari De Bellvitge	Xose PEREZ-FERNANDEZ	JOAN SABATER-RIERA
	UK	Royal Surrey NHS Foundation Trust	Lui Forni	
		University Hospitals of Derby and Burton NHS Foundation Trust	Nick Selby	Samantha Cole<br>Myint Lin<br>Victoria Wilkinson-Smith
		NHS Health Scotland	Samira Bell	Rachel Bittern
	Japan	The University of Tokyo Hospital	Kent Doi	Komaru,Yohei<br>Ryohei Horie
		National Center for Global Health and Medicine	Daisuke Katagiri	Noiri,Eisei
	Taiwan	Taipei Veterans General Hospital	Chiao-Lin Chuang	
		Taiwan University Hospital, Taipei	Vin-Cent Wu	Dow-Ming Huang
UMICs	Bolivia	Clinica Los Olivos	Rolando Claure-Del Granado	Mariscal, Andrea
		Hospital Obrero	Rolando Claure-Del Granado	Romero, Ximena
	Brazil	Hospital São Rafael – Rede D'Or São Luiz	Rogério Da Hora Passos	Fernanda Oliveira Coelho
		Rio de Janeiro State University	Elizabeth Maccariello	
	Dominican Republic	CEDIMAT	Guillermo Antonio Alvarez	Alberto Guarione Flore
	Mexico	Hospital Civil de Guadalajara Fray Antonio Alcalde	Guillermo Garcia-Garcia	Chavez-Iñiguez, Jonathan
	Peru	Hospital Nacional Arzobispo Loayza	Abdias Hurtado	Yanissa,Venega
	Russia	First Pavlov Saint- Petersburg State Medical University	Yury Polushi	Dmitry Sokolov
	Indonesia	RSPAD Gatot Soebroto Presidential Hospital	Jonny Lau	Dara Mega Agiana
	Malaysia	Hospital Kuala Lumpur	Nurul Zaynah Nordin	Bavanandan, Sunita
		Hospital Sungai Buloh	Eddie Wong Fook Sem	
		Hospital Sultanah Aminah, Johor Bahru	Liu Wen Jiun	Cheng Jin Kiang<br>Lee Soon Leng<br>Chong Ad Rian
		Hospital Enche' Besar Hajjah Khalsom,Kluang, Johor	Liu Wen Jiun	Noor Hidayah Yahya
		Hospital Permai, Johor Bahru	Liu Wen Jiun	Fariz Nordin<br>Kenneth Khoo Kay Leong<br>Choe Jing Ling

		Hospital Raja Permaisuri Bainun, Ipoh	Loh Chek Loong	Lee, Yee Yan
		Pusat Perubatan Universiti Kebangsaan Malaysia	Ruslinda Mustafar	Abdul Halim Abdul Gafor
	Thailand	King Chulalonkorn Memorial Hospital	Nattachai Srisawat	
LMICs	Egypt	Saudi German Hospital	Salem Aly Eldeeb	Hayat Mahmoud Mahmoud
	India	Gandhi Medical College	Manjusha Yedla	
		Jawaharlal Institute of Postgraduate Medical Education & Research	Sreejith Parameswaran	
	Nepal	B P Koirala Institute of Health Sciences	Sanjib Kumar Sharma	Mamit Rai

Abbreviations: HICs, high income countries; UMICs, upper middle income countries; LMICs, lower middle income countries

eTable 2. Electronic case report forms

: separately provided

eTable 3. Definitions

Time of assessment	Terminology	Definition
At admission	Baseline serum creatinine	Latest measured serum creatinine in the last 3-12 months prior to this hospital admission
	No Kidney Disease (NKD)	No Known diagnosis of CKD; eGFR at admission ≥60 mL/min/1.73 m2
	Acute Kidney Disease (AKD)	No Known diagnosis of CKD; eGFR at admission <60 mL/min/1.73 m2
	Chronic Kidney disease (CKD)	Known diagnosis of CKD; Prior evidence of markers of kidney damage for ≥3 months (microalbuminuria, proteinuria >300mg/24 hrs or abnormalities in imaging tests) or the presence of glomerular filtration rate (GFR) <60 mL/min/1.73 m2 for ≥3 months calculated with CKD-EPI equation, with or without other signs of kidney damage as described above.
	End-Stage Kidney Disease (ESKD)	Known diagnosis of ESKD on maintenance dialysis for more than 3 months
During hospital admission	Reference serum creatinine	Serum creatinine level at hospital admission
	Acute kidney injury (AKI)	An abrupt increase or decrease in serum creatinine level by 0.3 mg/dL within 48 hours or,<br>An abrupt increase or decreased in serum creatinine level by 50% from baseline within 7 days or,<br>Documented oliguria less than 0.3 ml/kg/hr for 6 hours of longer
	AKI type	
	De novo AKI	Evidence of AKI without prior diagnosis of CKD
	AKI on NKD	Evidence of AKI with criteria of NKD
	AKI on AKD	Evidence of AKI with criteria of AKD
	AKI on CKD	Evidence of AKI with criteria of kidney damage as stated with CKD definition
	AKI on KT	Evidence of AKI with criteria in kidney transplant recipient
	AKI developing time	
	Community acquired AKI	AKI developed within 48 hours of hospital admission
	Hospital acquired AKI	AKI developed after48 hours of hospital admission
	AKI severity	
	AKI stage 1	A 1.5-1.9 fold increase in serum creatinine from baseline
	AKI stage 2	A 2-2.9-fold increase in serum creatinine from baseline
	AKI stage 3	A three-fold or greater increase in serum creatinine from baseline or a peak serum creatinine ≥ 4.0 mg/dl<br>Patients with AKI treated with KRT were not included, as AKI III -KRT was analyzed as a separate category.

	AKI stage 3D	AKI treated with KRT at any time during hospital admission
At discharge	Combined outcome	Death or KRT dependence at hospital discharge
At discharge (Among survivors)	AKI	
	Complete recovery	Serum creatinine returned to baseline or lower
	Partial recovery	Serum creatinine lower than peak but higher than baseline
	Non-recovery	Dialysis dependence at discharge
	CKD/KT recipients	
	CKD progression	Creatinine change in percentage is >150% or requiring dialysis 72 hours prior to discharge.

Abbreviations: NKD, no kidney disease; AKD, acute kidney disease; ESKD, end stage kidney disease; AKI, acute kidney injury; KT, kidney transplantation; KRT, kidney replacement therapy

eTable 4. Number of missing information for collected Data

	Number of data, available (N,%) for all patients	Number of data, available (N,%) for AKI patients
**Demographics**		
Age, years (median, IQR)	4158(100)	3038(100)
Age, years (Mean±SD)	4158(100)	3038(100)
Male, n(%)	4158(100)	3038(100)
Race, n(%)	4158(100)	3038(100)
White	4158(100)	3038(100)
Black	4158(100)	3038(100)
Hispanic or Latino	4158(100)	3038(100)
Asian	4158(100)	3038(100)
Others	4158(100)	3038(100)
Continent, n(%)	4158(100)	3038(100)
North America	4158(100)	3038(100)
South America	4158(100)	3038(100)
Europe	4158(100)	3038(100)
Asia	4158(100)	3038(100)
National Income		
HICs	4158(100)	3038(100)
UMICs	4158(100)	3038(100)
LMICs	4158(100)	3038(100)
Weight, kg (Median, IQR)	2840(68.3)	2257(74.3)
Height, cm (Mean±SD)	2568(61.8)	2149(70.7)
BMI		
Male, kg/m2 (median, IQR)	1614(61.2)	1319(68.5)
Female, kg/m2 (median, IQR)	1012(66.6)	808(72.7)
**Kidney health status**		
AKI on NKD	4158(100)	3038(100)
AKI on AKD	4158(100)	3038(100)
AKI on CKD	4158(100)	3038(100)
AKI on KT	4158(100)	3038(100)
CKD without AKI	4158(100)	3038(100)
KT without AKI	4158(100)	3038(100)
ESKD	4158(100)	3038(100)

**Comorbidities, n(%)**		
Hypertension	4140(99.6)	3021(99.4)
Diabetes	4123(99.2)	3004(98.9)
Cardiovascular disease	4117(99.0)	3000(98.7)
Lung disease	4113(98.9)	2998(98.7)
Congestive heart failure	4088(98.3)	2976(98.0)
Active malignancy	4141(99.6)	3022(99.5)
Asthma	4113(98.9)	2995(98.6)
Other immunodeficiency	4068(97.8)	2959(97.4)
Liver disease	4115(99.0)	2999(98.7)
HIV infection	4145(99.7)	3026(99.6)
Total Numbers of Comorbidities (median, IQR)	4028(96.9)	2920(96.2)
Smoking (n, %)		
Non smoker	3771(90.7)	2740(90.2)
Former smoker	3771(90.7)	2740(90.2)
Current smoker	3771(90.7)	2740(90.2)
Unknown	3771(90.7)	2740(90.2)
**Home medication, n(%)**		
Statin	4040(97.2)	2923(96.2)
Beta blocker	4031(96.9)	2918(96.1)
Diuretics	4032(97.0)	2916(96.0)
ACEI	4006(96.3)	2900(95.5)
ARB	4017(96.6)	2902(95.5)
Anticoagulant agent	4016(97.0)	2903(95.6)
Immunosuppressant	4022(96.7)	2912(95.9)
NSAID	4011(96.5)	2900(95.5)
Herbal Medication	3989(95.9)	2880(94.8)
Total Numbers of Medications (median, IQR)	3989(95.9)	2847(93.7)
Enrollment at any investigational trial		
Yes,	3775(90.8)	2721(89.6)
**Reasons for Hospital admission, n(%)**		
Respiratory diagnosis	4134(99.4)	3019(99.4)
Infection	4101(98.6)	2988(98.4)
Worsening renal dysfunction	4141(99.6)	3022(99.5)
Metabolic issues	4099(98.6)	2984(98.2)
Sepsis	4143(99.6)	3026(99.6)

Shock/Hemodynamic instability	4127(99.3)	3010(99.1)
Heart diagnosis	4113(98.9)	2995(98.6)
Central nervous system	4119(99.1)	3003(98.8)
Trauma	4148(99.8)	3029(99.7)
Post-surgical	4155(99.9)	3036(99.9)
Others	3881(93.3)	2781(91.5)
**AKI diagnosis criteria**		
Oliguria	NA	2166(71.3)
Increasing serum creatinine criteria	NA	2166(71.3)
Decreasing serum creatinine criteria	NA	2166(71.3)
**AKI developing time**		
Community acquired (<48hr)	NA	2813(92.6)
Hospital acquired (≥48hr)	NA	2813(92.6)
**Volume status at admission, n(%)**		
Euvolemic	NA	3038(100)
Dehydrated	NA	3038(100)
Overloaded	NA	3038(100)
**AKI Severity at diagnosis**		
Stage 1, %	NA	2607(85.8)
Stage 2, %	NA	2607(85.8)
Stage 3, %	NA	2607(85.8)
Stage 3D, %	NA	2607(85.8)
**AKI Severity at peak**		
Peak Scr, mg/dL, median(IQR)	NA	2830(93.2)
Stage 1, %	NA	2830(93.2)
Stage 2, %	NA	2830(93.2)
Stage 3, %	NA	2830(93.2)
Stage 3D, %	NA	2830(93.2)
**Processes of care**		
Admission duration, days (median, IQR)	3843(92.4)	2822(94.9)
ICU care, yes(%)	4158(100)	3038(100)
ECMO, yes(%)	4090(98.4)	2977(98.0)
Vent care, yes(%)	4145(99.7)	3027(99.6)
Dialysis, yes(%)	4141(99.6)	3021(99.4)
Vent and Dialysis, N(%)	4136(99.5)	3018(99.3)
KRT modality Among KRT received patients(N=1797)		
IHD only	1797(99.7)	902(99.3)

CKRT only	1797(99.7)	902(99.3)
Both of IHD and CKRT	1797(99.7)	902(99.3)
Others (UF or PD)	1797(99.7)	902(99.3)
Enrolled at any kind of investigative trial, yes	3776(90.8)	2721(89.6)
**Patient Outcome**		
Survival status	3983(95.8)	2721(89.6)
Combined outcome	NA	2937(96.7)
**Among survivors**		
**Kidney outcome**		
AKI recovery		
Complete, %	NA	1663(98.5)
Partial, %	NA	1663(98.5)
Non-recovery, %	NA	1663(98.5)
CKD progression	1590(94.2)	1590(94.2)
**Discharge information among survivors**		
Discharge to, N(%)		
Home	2450(99.9)	1685(99.8)
Nursing Home	2450(99.9)	1685(99.8)
Other healthcare facility	2450(99.9)	1685(99.8)
Other	2450(99.9)	1685(99.8)

Abbreviations: HICs, high income countries; UMICs, upper middle income countries; LMICs, lower middle income countries; AKI, acute kidney injury; NKD, no kidney disease; AKD, acute kidney disease; ESKD, end stage kidney disease; KT, kidney transplantation; ACEI, angiotensin converting enzyme inhibitor; ARB, angiotensin receptor blocker; NSAID, non-steroidal anti-inflammatory drug; ICU, intensive care unit; ECMO, extracorporeal membrane oxygenation; Vent; ventilator; KRT, kidney replacement therapy; IHD, intermittent hemodialysis; CKRT, continuous kidney replacement therapy; UF, ultrafiltration; PD peritoneal dialysis

eTable 5. AKI characteristics and outcomes, stratified by national income status

	**HICs (N=1887)**				**UMICs (N=812)**				**LMICs (N=339)**			
	NKD (N=539)	AKD (N=725)	CKD (N=542)	KT (N=81)	NKD (N=355)	AKD (N=295)	CKD (N=151)	KT (N=11)	NKD (N=71)	AKD (N=153)	CKD (N=106)	KT (N=9)
**Demographics**												
Age, year	61(52, 71)	70(60, 80)	74(63, 83).	54(42, 66)	60(50,70)	67(57, 76)	67(57,76)	45(30, 58)	63(49,70)	60(45.5, 74)	57.5(48,70)	38(35,51)
Male, n(%)	394(73.1)[*§¶]	398(54.9)	331(61.1)	42(51.9)	241(67.9)	192(65.1)	99(65.6)	8(72.7)	51(71.8)	97(63.4)	69(65.1)	4(44.4)
Race, n(%)												
White	265(49.2)	355(49.0)	257(47.4)	30(37.0)	1(0.3)[¶]	5(1.7)[¶]	3(2.0)[¶]	2(18.2)	0	0	0	0
Black	85(15.8)[*§¶]	230(31.7)[¶]	170(31.4)	40(49.4)	1(0.3)	1(0.3)	4(2.6)	0	0	0	0	0
Hispanic or Latino	82(15.8)[*§]	56(7.7)	37(6.8)	10(12.3)	203(57.2)	166(56.3)	74(49.0)	5(45.5)	24(33.8)	41(26.8)	14(13.2)	0
Asian	58(10.8)[*§¶]	44(6.1)	37(6.8)	1(1.2)	146(41.1)	121(41.0)	66(43.7)	3(27.3)	47(66.2)	112(73.2)	92(86.8)	9(100)
Others	49(9.1)	40(5.5)	41(7.6)	0	4(1.1)	2(0.7)	4(2.6)	1(9.1)	0	0	0	0
**Comorbidities,** N(%)												
Hypertension	204(37.8)[*§¶]	401(55.3)[§]	348(64.2)	53(65.4)	190(54.4)[*§]	191(65.0)[§]	133(88.1)	6(54.5)	20(32.3)[§]	64(42.1)	75(70.8)	3(33.3)
Diabetes	144(26.7)[§]	235(32.4)[§]	246(45.4)	38(46.9)	137(40.2)[*§]	146(50.9)[§]	105(70.5)	3(27.3)	12(19.4)[*§]	61(40.1)	58(54.7)	1(11.1)
Cardiovascular disease	75(13.9)[*§]	142(19.6)[§]	175(32.3)	15(18.5)	38(11.3)[§]	47(16.2)	38(25.7)	0	7(11.3)	17(11.2)	13(12.3)	0
Lung disease	65(12.1)[§]	103(14.2)	106(19.6)	5(6.2)	45(13.2)	27(9.5)	17(11.7)	0	4(6.5)	8(5.3)	3(2.8)	0
Congestive heart failure	28(5.2)[§]	63(8.7)[§]	115(21.2)	6(7.4)	19(5.9)[§]	14(4.9)	15(10.4)	0	1(1.6)	2(1.3)	3(2.8)	0
Active malignancy	36(6.7)	65(9[*§¶].0)	51(9.4)	5(6.2)	25(7.1)	20(6.8)	10(6.7)	1(9.1)	1(1.6)	5(3.2)	2(1.9)	0
Asthma	31(5.8)	40(5.5)	29(5.4)	1(1.2)	24(7.1)[§]	9(3.2)	1(0.7)	0	4(6.3)	0	0	0
Other immunodeficiency	8(1.5)[§¶]	20(2.8)[§¶]	31(5.7)	7(8.6)	37(11.1)[¶]	17(5.9)[¶]	11(7.7)[¶]	5(45.5)	1(1.9)	1(0.7)	3(3.0)	0
Liver disease	17(3.2)	19(2.6)	29(5.4)	6(7.4)	12(3.5)	11(3.8)	8(5.5)	0	4(6.5)	4(2.6)	3(2.8)	0
HIV infection	4(0.7)	4(0.6)	3(0.6)	0	7(2.0)	2(0.7)	2(1.3)	0	0	1(0.7)	0	0
**Home medication,** N(%)												
Statin	114(21.2)[*§¶]	230(31.7)[§¶]	242(44.6)	35(43.2)	77(25.5)[§]	66(25.8)[§]	72(53.7)	3(30.0)	5(7.2)	20(13.3)	10(9.4)	0
Beta blocker	70(13.0)[*§¶]	183(25.2)[§¶]	210(38.7)	42(51.9)	56(18.8)[§]	46(18.1)	45(33.3)	4(40,0)	6(8.7)	3(2.0)	2(1.9)	0
Diuretics	63(11.7)[*§¶]	177(24.4)[¶]	194(35.8)	28(34.6)	33(11.0)[§]	32(12.7)	42(31.1)	1(10.0)	3(4.4)	5(3.3)	13(12.3)	0
ACEI	67(12.4)[§¶]	106(14.6)	100(18.5)[¶]	1(1.2)	61(20.4)	43(17.8)[§]	46(35.7)	4(40.0)	4(5.8)	4(2.7)	4(3.8)	0
ARB	56(10.4)[*§]	117(16.1)	93(17.2)	12(14.8)	53(18.0)	60(24.6)	29(22.0)	2(20.0)	4(5.8)	16(10.7)[§]	1(0.9)	0
Anticoagulant agent	51(9.5)[*§¶]	105(14.5)[§¶]	116(21.4)	8(9.9)	24(8.2)[§]	32(13.5)	22(17.1)	2(20.0)	2(2.9)	3(2.0)	1(0.9)	0
Immunosuppressant	20(3.7)[¶]	30(4.1)[¶]	40(7.4)[¶]	59(72.8)	35(11.8)[¶]	20(8.0)[¶]	17(12.9)[¶]	5(45.5)	1(1.4)	0	0	0
NSAID	31(5.8)	35(4.8)	51(9.4)	5(6.2)	23(7.9)	23(9.3)	11(8.3)	1(10.0)	8(11.6)	2(1.3)	1(0.9)	0

Herbal Medication	4(0.7)	1(0.1)	2(0.4)	1(1.2)	0	4(1.7)	0	0	1(1.4)	3(2.0)	1(0.9)	0
**Reasons for Hospital admission,** N(%)												
Respiratory diagnosis	346(64.2)*§¶	399(55.0)	298(55.0)	34(42.0)	271(79.5)	221(75.7)	108(72.5)	9(81.8)	58(81.7)§	101(66.0)	63(59.4)	6(66.7)
Infection	214(39.7)	267(36.8)	206(38.0)	33(40.7)	198(60.0)*	122(43.6)	77(54.6)	4(36.4)	42(59.2)	95(62.1)	42(39.6)	0
Worsening renal dysfunction	13(2.4) *§¶	205(38.3)	129(23.8)	28(34.6)	11(3.2) *§¶	63(21.9) §¶	54(36.5)	5(45.5)	18(25.4)*§	69(45.1)	56(52.8)	5(55.6)
Metabolic issues	22(4.1) *§	72(9.9)	48(8.9)	8(9.9)	40(12.6) *§	61(21.6)	43(29.1)	3(27.3)	14(19.7)	30(19.6)	23(21.7)	1(11.1)
Sepsis	67(12.4) *	134(18.5)	78(14.4)	9(11.1)	97(27.8)	73(25.2)	37(24.7)	1(9.1)	8(11.3)	22(14.4)	8(7.5)	0
Shock/Hemodynamic instability	36(6.7)	61(8.4)	38(7.0)	1(1.2)	45(13.4)	45(15.7)	15(10.1)	0	5(7.0)	22(14.4)	7(6.6)	0
Heart diagnosis	22(4.1) *§	89(12.3)	80(14.8)	7(8.6)	14(4.3)§	17(6.0)	16(11.0)	0	3(4.3)	7(4.6)	0	0
Central nervous system	11(2.0)	29(4.0)	25(4.6)	1(1.2)	7(2.1)	5(1.8)	3(2.1)	0	3(4.2)	5(3.3)	8(7.5)	0
Trauma	10(1.9)	12(1.7)	11(2.0)	0	2(0.6)	0	0	0	1(1.4)	1(0.7)	0	0
Post-surgical	6(1.1)	3(0.4)	1(0.2)	0	7(2.0)	7(2.4)	3(2.0)	1(9.1)	2(2.8)	3(2.0)	0	0
Others	60(11.1)*§¶	177(24.4)	129(23.8)	20(24.7)	9(4.0)	17(7.8)	8(7.8)	0	4(5.6)	9(5.9)	4(3.8)	0
**Volume status**, N(%)												
Dehydrated	120 (22.3)*§	334(46.1)§	207(38.2)	30(37.0)	33(9.3)	44(14.9)	25(16.6)	2(18.2)	17(23.9)	22(14.4)	10(9.4)	1(11.1)
Overloaded	47(8.7)	42(5.8)	50(9.2)	6(7.4)	20(5.6)§	24(8.1)	26(17.2)	2(18.2)	15(21.1)	48(31.4)	37(34.9))	1(11.1)
**AKI diagnosis criteria, N(%)**												
Oliguria	8(2.7) *§	58(12.4) §	24(6.5)	7(12.5)	13(5.1)	10(4.5)	11(8.9)	2(20.0)	1(1.5)	6(4.8)	4(4.9)	0
Increasing Cr criteria	204(68.5) *§	164(35.2) §	196(53.4)	25(44.6)	223(87.8)§	179(80.6)	95(76.6)	7(70.0)	45(68.2)§	71(57.3)	34(41.5)	6(66.7)
Decreasing Cr criteria	86(28.9) *§	244(52.4) §	147(40.1)	24(42.9)	198(7.1) *	33(14.9)	18(14.5)	1(10.0)	20(30.3) §	47(37.9)	44(53.7)	3(33.3)
**AKI developing time**, N(%)												
CA-AKI	215(47.8) *§	619(91.6) §	423(84.1)	70(87.5)	114(34.3) *§	178(63.6)	98(66.7)	8(72.7)	40(56.3) *	121(80.7)	65(62.5)	3(33.3)
HA-AKI	235(52.2) *§	57(8.4) §	80(15.9)	10(12.5)	218(65.7) *§	102(36.4)	49(33.3)	3(27.3)	31(43.7) *	29(19.3)	39(37.5)	6(66.7)
**AKI stage at diagnosis**, N(%)												
Stage1	315(78.4)	527(80.6)	396(81.3)	59(74.7)	131(45.5)*	150(63.8)	62(52.1)	3(30.0)	46(64.8)	93(62.8)	68(64.8)	3(33.3)
Stage 2	49(12.2)	62(9.5)	53(10.9)	10(12.7)	45(15.6)*§	12(5.1)	4(3.4)	1(10.0)	10(14.1)§	12(8.1)	4(3.8)	1(11.1)
Stage 3	18(4.5)	37(5.7)	27(5.5)	6(7.6)	28(9.7)§	12(5.1)	1(0.8)	0	9(12.7)	9(6.1)	2(1.9)	1(11.1)
Stage 3-KRT,	20(5.0)	28(4.3)	11(2.3)	4(5.1)	84(29.2)§	61(26.0)	52(43.7)	6(60.0)	6(8.5)§	34(23.0)	31(29.5)	4(44.4)
**Peak AKI stage**												
Stage 1	257(53.1)*§	457(65.7)	341(64.7)	44(54.3)	68(21.2)	77(30.3)	28(20.3)	2(20.0)	31(47.0)	51(36.2)	47(45.6)	2(22.2)
Stage 2	69(14.3)	75(10.8)	71(13.5)	12(14.8)	27(8.4)	16(6.3)	3(2.2)	1(10.0)	15(22.7)§	20(14.2)	5(4.9)	1(11.1)
Stage 3	54(11.2)§	48(6.9)	30(5.7)	3(3.7)	24(7.5) *	6(2.4)	2(1.4)	0	8(12.1)§	21(14.9)	2(1.9)	1(11.1)
Stage 3-KRT	102(21.1)	116(16.7)	85(16.1)	22(27.2)	202(62.9)§	155(61.0)	105(76.1)	7(70.0)	11(16.7)*§¶	49(34.8)	49(47.6)	5(55.6)
**Process of care**, N(%)												
ICU care	345(64.0)*§	324(44.7)§	173(31.9)	32(39.5)	251(70.7) *	171(58.0)	93(61.6)	5(45.5)	46(64.8)	98(64.1)	52(49.1)	3(33.3)
ECMO	35(6.7)§	33(4.6)	4(0.8)	1(1.3)	31(9.0)	30(10.5)	18(11.9)	1(9.1)	2(2.8)	2(1.3)	1(1.0)	0

Vent	316(59.2)*§	253(34.9)§	133(24.7)	25(30.9)	282(79.7)*	175(59.5)	88(58.3)	6(54.5)	28(39.4)	46(30.1)	55(23.8)	2(22.2)
KRT	102(19.2)	116(16.1)	85(15.8)	22(27.2)	202(57.1)	155(52.7)§	105(69.5)	7(63.6)	11(15.1)§¶	49(32.0)	49(46.2)	5(55.6)
Vent and KRT	102(19.2)	108(15.0)	68(12.7)	16(19.8)	201(56.8)	130(44.2)	82(54.3)	6(54.5)	10(14.1)	23(15.0)	19(18.1)	1(11.1)
KRT modality among KRT received patients												
IHD only	8(7.8)	4(3.4)	14(17.3)	5(23.8)	61(30.2)	72(46.5)	50(47.5)	50(47.6)	9(81.8)	40(83.3)	46(93.9)	4(80.0)
CKRT only	63(61.8)	57(49.1)	42(51.9)	9(42.9)	86(42.6)	38(24.55)	25(23.8)	0	2(18.2)	2(4.2)	0	0
Both of IHD and CKRT	5(4.9)	20(17.2)	7(8.6)	2(9.5)	52(25.7)	41(26.5)	29(27.6)	3(42.9)	0	0	0	0
Others (UF or PD)	26(25.5)	35(30.2)	18(22.2)	5(23.8)	3(1.5)	4(2.6)	1(1.0)	0	0	2(12.5)	3(6.1)	1(20.0)
**Enrollment at any investigational trial, N(%)**												
Yes	103(21.8)*	83(12.1)	80(15.7)	11(14.9)	4(1.3)	2(0.8)	1(0.8)	0	1(1.6)	0	0	0
**Patient outcome**, N(%)												
Death	196(38.1)¶	241(33.9)	190(35.6)	18(22.2)	203(59.5)	164(57.5)	82(56.9)	3(30.0)	35(53.0)	76(54.7)§	38(37.3)	3(33.3)
**Renal outcome**, N(%)												
Complete	288(53.4)§¶	386(53.2)	163(30.1)	29(35.8)	79(22.3)§	82(27.8)§	28(18.5)	4(36.4)	22(31.0)	33(21.6)	5(4.7)	0
Partial	101(18.7)*§	188(25.9)§	237(43.7)	28(34.6)	102(28.7)	73(24.7)	33(21.9)	1(9.1)	10(14.1)§	30(19.6)	52(49.1)	4(44.4)
Non-recovery	76(14.1)¶	90(12.4)¶	81(14.9)¶	23(28.4)	159(44.8)	124(42.0)	80(53.0)	5(45.5)	22(31.0)	52(34.0)	30(28.3)	4(44.4)
**Combined outcome (Death or RRT dependence at discharge)**, N(%)												
YES	158(34.0)	216(32.5)	162(33.7)	29(36.3)	212(62.4)	168(60.2)	88(62.4)	5(50.0)	25(46.3)	67(58.3)§	34(39.1)	4(50.0)

Abbreviations: HICs, high income countries; UMICs, upper middle income countries; LMICs, lower middle income countries; AKI, acute kidney injury; NKD, no kidney disease; AKD, acute kidney disease; CKD, chronic kidney disease; KT, kidney transplantation; ACEI, angiotensin converting enzyme inhibitor; ARB, angiotensin receptor blocker; NSAID, non-steroidal anti-inflammatory drug; Cr, creatinine; CA-AKI, community acquired AKI; HA-AKI, hospital acquired AKI; KRT, kidney replacement therapy; ICU, intensive care unit; ECMO, extracorporeal membrane oxygenation; Vent; ventilator; IHD, intermittent hemodialysis; CKRT, continuous kidney replacement therapy; UF, ultrafiltration; PD peritoneal dialysis

Foot note: *p<0.05 Compared to AKD, § p<0.05 Compared to CKD, ¶ p<0.05 Compared to KT
Data regarding age were missing for 0 patients (0%).
Data regarding male were missing for 0 patients (0%).
Data regarding race were missing for 0 patients (0%).
Data regarding hypertension were missing for 17 patients (0.6%).
Data regarding diabetes were missing for 34 patients (1.1%).
Data regarding CVD were missing for 38 patients (1.3%).
Data regarding lung disease were missing for 40 patients (1.3%).
Data regarding CHF were missing for 62 patients (2.0%).
Data regarding cancer were missing for 16 patients (0.5%).
Data regarding asthma were missing for 43 patients (1.4%).
Data regarding IMD were missing for 79 patients (2.6%).
Data regarding liver disease were missing for 39 patients (1.3%).
Data regarding HIV were missing for 12 patients (0.4%).
Data regarding statin were missing for 115 patients (3.8%).
Data regarding beta blocker were missing for 120 patients (3.9%).
Data regarding diuretics were missing for 122 patients (4.0%).
Data regarding ACEI were missing for 138 patients (4.5%).
Data regarding ARB were missing for 136 patients (4.5%).

Data regarding anticoagulation were missing for 135 patients (4.4%).
Data regarding IMD were missing for 126 patients (4.1%).
Data regarding NSAID were missing for 138 patients (4.5%).
Data regarding herbal medication were missing for 158 patients (5.2%).
Data regarding respiratory diagnosis were missing for 19 patients (0.6%).
Data regarding infection were missing for 50 patients (1.6%).
Data regarding worsening of renal function were missing for 16 patients (0.5%).
Data regarding metabolic disease were missing for 54 patients (1.8%).
Data regarding sepsis were missing for 12 patients (0.4%).
Data regarding shock were missing for 28 patients (0.9%).
Data regarding cardiac disease were missing for 43 patients (1.4%).
Data regarding CNS were missing for 35 patients (1.2%).
Data regarding trauma were missing for 9 patients (0.3%).
Data regarding post OP were missing for 2 patients (0.1%).
Data regarding other causes were missing for 257 patients (8.5%).
Data regarding volume status were missing for 0 patients (0%).
Data regarding oliguria were missing for 12 patients (0.4%)
Data regarding AKI developing time were missing for 225 patients (7.4%)
Data regarding AKI stage at diagnosis were missing for 431 patients (14.2%).
Data regarding AKI stage at peak were missing for 208 patients (6.8%).
Data regarding ICU care were missing for 0 patients (0%)
Data regarding ECMO were missing for 61 patients (2.0%)
Data regarding Ventilator care were missing for 11 patients (0.4%)
Data regarding RRT were missing for 17 patients (0.6%)
Data regarding vent and RRT were missing for 20 patients (0.7%)
Data regarding KRT modality among KRT received patients were missing for 6 patients (0.7%)
Data regarding enrollment at any investigational trial were missing for 317 patients (%).
Data regarding death were missing for 101 patients (3.3%).
Data regarding kidney recovery were missing for 0 patients (0%).
Data regarding combined outcome were missing for 314 patients (10.3%).

eTable 6. Multivariable binary regression model predicting in-hospital mortality in all patients

	Unadjusted Model		Final Model	
	OR	95%(CI)	OR	95%(CI)
**Age**				
< 55 years	Reference		Reference	
55≤ age <65	1.371	1.137, 1.653	1.062	0.826, 1.365
65≤ age <75	2.054	1.718, 2.457	2.386	1.857, 3.064
≥ 75 years	2.267	1.889, 2.721	5.165	3.890, 6.859
Male	1.228	1.074, 1.403	-	
**Race**				
White	Reference		Reference	
Black	0.561	0.450, 0.700	0.555	0.422, 0.729
Hispanic or Latino	1.724	1.435, 2.072	0.953	0.686, 1.324
Asian	1.020	0.861, 1.208	1.060	0.747, 1.504
Other races	0.739	0.520, 1.049	0.705	0.455, 1.090
**Comorbidities**				
Diabetes	1.188	1.044, 1.353	-	
Hypertension	1.137	0.997, 1.297	1.205	0.997, 1.457
Congestive heart failure	1.257	0.998, 1.584	1.748	1.278, 2.390
Liver disease	1.118	0.798, 1.567	1.668	1.046, 2.660
Lung disease	1.198	0.980, 1.465	-	
**National Income**				
HICs	Reference		Reference	
UMICs	1.900	1.638, 2.203	1.267	0.904, 1.775
LMICs	1.471	1.236, 1.751	1.481	0.996, 2.201
**Medications**				
ACEI/ARBs	0.963	0.829, 1.118	-	
Anticoagulants	1.260	1.031, 1.540	-	
Statin, Yes	0.864	0.748, 0.998	0.696	0.563, 0.860
Investigational trial drug, Yes	0.559	0.437, 0.716	0.430	0.315, 0.586
**Severity of disease**				
Shock at admission, Yes	3.494	2.757, 4.427	2.075	1.525, 2.822

ICU admission, Yes	7.569	6.548, 8.749	2.684	2.106, 3.420
**Process of care**				
Neither ventilator nor KRT	Reference		Reference	
Ventilator care alone	4.218	3.457, 5.146	3.624	2.678, 4.905
KRT alone	0.807	0.650, 1.002	1.116	0.775, 1.608
Both ventilator and KRT	11.466	9.476, 13.873	9.118	6.555, 12.682
**Kidney function status**				
ESKD	Reference		Reference	
No AKI	0.407	0.273, 0.607	0.531	0.316, 0.893
AKI	1.726	1.462, 2.037	1.099	0.795, 1.519
**National Income X Process of care**				
HICs and Neither ventilator nor KRT			Reference	
UMICs and ventilator care alone			2.675	1.320,5.419
UMICs and KRT alone			1.072	0.516, 2.226
UMICs and both ventilator and KRT			0.735	0.400, 1.349
LMICs and ventilator care alone			6.550	2.428, 17.668
LMICs and KRT alone			3.200	1.418, 7.220
LMICs and both ventilator and			6.209	2.606, 14.793

Final model was adjusted with age, race, hypertension, congestive heart failure, liver disease, national income, statin, investigational drug, shock at admission, ICU use, processes of care, kidney health status, and interaction between process of care and national income status

Abbreviations: HICs, high income countries; UMICs, upper middle income countries; LMICs, lower middle income countries; ACEI, angiotensin converting enzyme inhibitor; ARB, angiotensin receptor blocker; ICU, intensive care unit; KRT, kidney replacement therapy; ESKD, end stage kidney disease; AKI, acute kidney injury

eTable 7. Multivariable binary regression models predicting in-hospital mortality and combined outcome in AKI patients

	In-hospital mortality		Combined outcome	
	Unadjusted Model	Final Model*	Unadjusted Model	Final Model**
	OR(95% CI)	OR(95%CI)	OR(95% CI)	OR(95%CI)
Age				
< 55 years	Reference	Reference	Reference	Reference
55≤ age <65	1.273(1.016, 1.592)	0.884(0.648, 1.205)	1.325(1.058, 1.658)	1.022(0.725, 1.442)
65≤ age <75	1.837(1.487, 2.270)	1.865(1.371, 2.535)	1.590(1.282, 1.972)	1.399(0.992, 1.973)
≥ 75 years	2.210(1.787, 2.732)	5.295(3.774, 7.429)	1.676(1.347, 2.086)	4.058(2.753, 5.982)
Male	1.241(1.066, 1.446)	-	1.179 (1.006, 1.380)	-
Race				
White	Reference	Reference	Reference	Reference
Black	0.545(0.431, 0.689)	0.530(0.394, 0.712)	0.763(0.603, 0.965)	0.767(0.533, 1.019)
Hispanic or Latino	1.989(1.627, 2.431)	1.177(0.816, 1.696)	2.778(2.248, 3.434)	0.877(0.557, 1.381)
Asian	1.257(1.027, 1.538)	1.256(0.853, 1.850)	1.329(1.0072, 1.648)	0.837(0.503, 1.393)
Other races	0.841(0.427, 1.655)	0.929(0.563, 1.530)	0.942(0.628, 1.414)	0.687(0.379, 1.247)
**Comorbidities**				
Diabetes	1.182(1.017, 1.374)	-	1.161(0.993, 1.357)	-
Hypertension	1.289(1.112, 1.496)	-	1.357(1.163, 1.583)	-
Congestive heart failure	1.237(0.958, 1.597)	1.767(1.239, 2.521)	1.137(1.869, 1.488)	1.796(1.196, 2.697)
Liver disease	1.197(0.820, 1.748)	1.741(1.004, 3.017)	1.487(1.008, 2.194)	1.640(0.874, 3.079)
Lung disease	1.219(0.980, 1.517)	0.907(0.658, 1.251)	1.195(0.951, 1.502)	-
**National Income**				
HICs	Reference	Reference	Reference	Reference
UMICs	2.555(2.153, 3.033)	0.795(0.541, 1.166)	3.171(2.657, 3.784)	1.099(0.679, 1.779)
LMICs	1.719(1.351, 2.186)	1.089(0.668, 1.774)	1.932(1.487, 2.509)	1.140(0.617, 2.106)
**Medications**				
ACEI/ARBs	1.035(0.875, 1.225)	-	1.015(0.853, 1.208)	-
Anticoagulants	1.246(0.998, 1.555)	-	1.172(0.929, 1.478)	-
Statin, Yes	0.963(0.818, 1.134)	0.704(0.556, 0.890)	1.055 (0.895, 1.249)	0.723(0.554, 0.944)
Investigational trial drug, Yes	0.510 (0.390, 0.668)	0.437(0.309, 0.617)	0.620(0.476, 0.808)	0.795(0.541, 1.169)
**AKI developing time**				
CA-AKI	Reference	-	Reference	Reference
HA-AKI	2.046(1.734, 2.414)	-	1.875(1.579, 2.226)	1.235(0.920, 1.659)

**Severity of AKI**				
Peak AKI stage		-		
Stage1	Reference	Reference	Reference	Reference
Stage2	3.010(2.335, 3.880)	2.653(1.920, 3.665)	3.276(2.489, 4.313)	2.601(1.807, 3.744)
Stage3	6.405(5.367, 7.643)	3.255(2.337, 4.737)	11.040(9.088, 13.411)	3.516(2.285, 5.408)
**Process of care**				
Neither ventilator nor KRT	Reference	Reference	Reference	Reference
Ventilator care alone	3.778(3.085, 4.628)	7.117(5.307, 9.543)	4.166(3.353, 5.178)	8.054(5.761, 11.260)
KRT alone	1.060(0.703, 1.598)	0.758(0.415, 1.384)	4.398(3.305, 6.373)	3.405(1.858, 6.241)
Both ventilator and KRT	10.270(8.350, 12.633)	9.723(6.273, 15.072)	18.106(14.360, 22.831)	16.578(10.031, 27.398)
**Baseline Kidney function status**				
NKD	Reference	Reference	Reference	Reference
AKD	0.827(0.694, 0.985)	1.244(0.953, 1.623)	0.873(0.728, 1.046)	1.502(1.0736, 2.102)
CKD	0.742(0.611, 0.900)	1.270(0.939, 1.717)	0.785(0.642, 0.960)	1.604(1.108, 2.322)
KT	0.355(0.220, 0.572)	0.878(0.471, 1.637)	0.744(0.485, 1.141)	2.144(1.116, 4.117)
**Process of care X National income**				
HICs X Neither ventilator nor KRT		Reference		Reference
UMICs X Ventilator care alone		2.852(1.187, 6.854)		1.787(0.723,4.417)
UMICs X KRT alone		1.001(0.198, 5.051)		0.065(0.016, 0.267)
UMICs X both ventilator and KRT		0.909(0.429, 1.925)		0.359(0.162, 0.793)
LMICs X Ventilator care alone		6.321(2.148, 18.601)		2.943(0.860, 10.062)
LMICs X KRT alone		6.831(1.639, 28.473)		0.505(0.130, 1.956)
LMICs X both ventilator and KRT		5.086(1.517, 17.059)		3.120(0.575, 16.934)

*Final model for inhospital mortality was adjusted with age, race, national income status, congestive heart failure, liver disease, lung disease, statin, investigational drug, AKI stage, process of care, and baseline kidney function status, and interaction of national income status X process of care.

and interaction of national income status X process of care

** Final modal for combine outcome was adjusted with age, race, congestive heart failure, liver disease, national income, statin, investigational drug, AKI developing time, AKI stage at peak, processes of care, and baseline kidney function status

Abbreviations: HICs, high income countries; UMICs, upper middle income countries; LMICs, lower middle income countries; ACEI, angiotensin converting enzyme inhibitor; ARB, angiotensin receptor blocker; CA-AKI, community acquired AKI; HA-AKI, hospital acquired AKI; AKI, acute kidney injury; KRT, kidney replacement therapy; NKD, no kidney disease; AKD, acute kidney disease; CKD, chronic kidney disease; KT, kidney transplantation;

eTable 8. In-hospital mortality by national income among AKI patients

	In-hospital mortality		Combined outcome
National Income		National Income	
HICs	Reference	HICs	Reference
UMICs	2.555(2.153, 3.033)	UMICs	3.171(2.657, 3.784)
LMICs	1.719(1.351, 2.186)	LMICs	1.932(1.487, 2.509)
National Income + Age		National Income + Age	
HICs	Reference	HICs	Reference
UMICs	2.479(2.086, 2.947)	UMICs	3.398(2.837, 4.070)
LMICs	1.710(1.344, 2.176)	LMICs	2.207(1.689, 2.884)
National Income + Age, Sex		National Income + Age, Race	
HICs	Reference	HICs	Reference
UMICs	2.465(2.073, 2.930)	UMICs	3.455(2.606, 4.580)
LMICs	1.701(1.336, 2.165)	LMICs	2.690(1.878, 3.852)
National Income + Age, Sex, Race		National Income + Age, Race, CHF, Liver Disease	
HICs	Reference	HICs	Reference
UMICs	2.333(1.798, 3.026)	UMICs	3.309(2.489, 4.399)
LMICs	1.858(1.341, 2.575)	LMICs	2.791(1.938, 4.020)
National Income + Age, Sex, Race, CHF, Liver Disease		National Income + Age, Race, CHF, Liver Disease, AKI developing time	
HICs	Reference	HICs	Reference
UMICs	2.302(1.769, 2.997)	UMICs	3.668(2.661,5.058)
LMICs	1.969(1.412, 2.745)	LMICs	3.515(2.362, 5.231)
National Income + Age, Sex, Race, CHF, Liver Disease, **AKI stage**		National Income + Age, Sex, Race, CHF, Liver Disease, AKI developing time, AKI stage	
HICs	Reference	HICs	Reference
UMICs	1.202(0.892, 1.68)	UMICs	1.686(1.157, 2.457)
LMICs	1.353(0.943, 1.941)	LMICs	2.490(1.587, 3.905)

National Income + Age, Sex, Race, CHF, Liver Disease, AKI stage, **process of care**		National Income +Age, Sex, Race, CHF, Liver Disease, AKI developing time, AKI stage, process of care, baseline kidney health status	
HICs	Reference	HICs	Reference
UMICs	1.138(0.826, 1.568)	UMICs	1.540(1.023, 2.321)
LMICs	2.268(1.547, 3.325)	LMICs	4.601(2.829, 7.494)
National Income + Age, Sex, Race, CHF, Liver Disease, AKI stage, process of care+ **investigational trial**		National Income+ Age, Sex, Race, CHF, Liver Disease, AKI developing time, AKI stage, process of care, baseline kidney health status, **statin, investigational trial**	
HICs	Reference	HICs	Reference
UMICs	0.795(0.541, 1.166)	UMICs	1.099(0.679, 1.779)
LMICs	1.089(0.668, 1.774)	LMICs	1.140(0.617, 2.106)

Abbreviations: AKI, acute kidney injury; HICs, high income countries; UMICs, upper middle income countries; LMICs, lower middle income countries; CHF, congestive heart failure;

eTable 9. Factors associated with partial or non-renal recovery among AKI survivors

	Unadjusted Model	Final Model
	OR(95% CI)	HR(95% CI)
Age		
< 55 years	Reference	-
55≤ age <65	1.026(0.783, 1.345)	
65≤ age <75	1.005(0.767, 1.315)	
≥ 75 years	0.966(0.731, 1.276)	
Male	0.944(0.773, 1.154)	-
Race		
White	Reference	Reference
Black	1.658(1.261, 2.179)	1.352(0.989, 1.849)
Hispanic or Latino	1.938(1.447, 2.594)	0.994(0.625, 1.581)
Asian	1.761(1.336, 2.321)	0.681(0.411, 1.129)
Other races	1.773(1.114, 2.822)	1.769(0.992, 3.153)
National Income		
HICs	Reference	Reference
UMICs	1.610(1.256, 2.064)	1.31(0.752, 2.015)
LMICs	3.722(2.641, 5.245)	4.158(2.392, 7.227)
AKI developing time		
CA-AKI	Reference	Reference
HA-AKI	1.321(1.047, 1.665)	1.420(1.043, 1.931)
Peak AKI stage		
Stage 1	Reference	Reference
Stage 2	1.897(1.358, 2.651)	2.065(1.428, 2.985)
Stage 3	2.890(1.850, 4.517)	3.735(2.273, 6.138)
Stage 3-KRT	7.215(5.356, 9.719)	7.517(5.176, 10.916)
Baseline kidney function status		

NKD	Reference	Reference
AKD	1.170(0.901, 1.520)	1.286(0.921, 1.795)
CKD	4.319(3.286, 5.676)	5.519(3.912, 7.786)
KT	3.588(2.185, 5.893)	4.044(2.291, 7.139)

Final model was adjusted with race, national income, AKI developing time, AKI stage at peak, and baseline kidney function status

Abbreviations: AKI, acute kidney injury; HICs, high income countries; UMICs, upper middle income countries; LMICs, lower middle income countries; CA-AKI, community acquired AKI; HA-AKI, hospital acquired AKI; KRT, kidney replacement therapy; NKD, no kidney disease; AKD, acute kidney disease; CKD, chronic kidney disease; KT, kidney transplantation;
